# Sustaining yield and nutritional quality of peanuts in harsh environments: Physiological and molecular basis of drought and heat stress tolerance

**DOI:** 10.3389/fgene.2023.1121462

**Published:** 2023-03-08

**Authors:** Naveen Puppala, Spurthi N. Nayak, Alvaro Sanz-Saez, Charles Chen, Mura Jyostna Devi, Nivedita Nivedita, Yin Bao, Guohao He, Sy M. Traore, David A. Wright, Manish K. Pandey, Vinay Sharma

**Affiliations:** ^1^ Agricultural Science Center at Clovis, New Mexico State University, Las Cruces, NM, United States; ^2^ Department of Biotechnology, University of Agricultural Sciences, Dharwad, India; ^3^ Department of Crop, Soil and Environmental Sciences, Auburn University, Auburn, AL, United States; ^4^ USDA-ARS Vegetable Crops Research, Madison, WI, United States; ^5^ Department of Horticulture, University of Wisconsin-Madison, Madison, WI, United States; ^6^ Biosystems Engineering Department, Auburn University, Auburn, AL, United States; ^7^ Department of Plant and Soil Sciences, Tuskegee University, Tuskegee, AL, United States; ^8^ Department of Biotechnology, Iowa State University, Ames, IA, United States; ^9^ International Crops Research Institute for the Semi-Arid Tropics (ICRISAT), Patancheru, Telangana, India

**Keywords:** drought, heat stress, physiological, molecular, high-throughput phenotyping, introgression, transgenes and CRISPR

## Abstract

Climate change is significantly impacting agricultural production worldwide. Peanuts provide food and nutritional security to millions of people across the globe because of its high nutritive values. Drought and heat stress alone or in combination cause substantial yield losses to peanut production. The stress, in addition, adversely impact nutritional quality. Peanuts exposed to drought stress at reproductive stage are prone to aflatoxin contamination, which imposes a restriction on use of peanuts as health food and also adversely impact peanut trade. A comprehensive understanding of the impact of drought and heat stress at physiological and molecular levels may accelerate the development of stress tolerant productive peanut cultivars adapted to a given production system. Significant progress has been achieved towards the characterization of germplasm for drought and heat stress tolerance, unlocking the physiological and molecular basis of stress tolerance, identifying significant marker-trait associations as well major QTLs and candidate genes associated with drought tolerance, which after validation may be deployed to initiate marker-assisted breeding for abiotic stress adaptation in peanut. The proof of concept about the use of transgenic technology to add value to peanuts has been demonstrated. Advances in phenomics and artificial intelligence to accelerate the timely and cost-effective collection of phenotyping data in large germplasm/breeding populations have also been discussed. Greater focus is needed to accelerate research on heat stress tolerance in peanut. A suits of technological innovations are now available in the breeders toolbox to enhance productivity and nutritional quality of peanuts in harsh environments. A holistic breeding approach that considers drought and heat-tolerant traits to simultaneously address both stresses could be a successful strategy to produce climate-resilient peanut genotypes with improved nutritional quality.

## Introduction

Climate change and hot weather extremes have perpetuated vulnerability in the ecosystem and agriculture sector, threatening food and nutritional security. Intergovernmental Panel on Climate Change (IPCC) assessment has estimated a 1.5°C rise in global warming in the near term (2021–2040). At the projected rising temperature of 2°C–3°C, the subsequent increase in frequency and severity of water scarcity (drought stress) will lead to severe loss in biodiversity and crop production in various geographic regions (IPCC, 2022). Drought is the single greatest abiotic stress, reducing yield under rainfed and irrigated cropping systems (Boyer, 1982; Araus et al., 2002). Drought produces the reduction of transpiration and thus photosynthesis which results in decreased biomass accumulation and yield (Tardieu and Tuberosa, 2010). For example, in 2012, the drought that happend in the United States (US) during summer and fall, cost approximately 30 billion dollars to the US economy (Riley, 2015). Additionally the US peanut industry losses every year 50 million dollars due to drought stress (U.S. Department of Agriculture and Agricultural Research Service, 2019).

Peanut is an important oilseed crop, widely grown across continents in semi-arid tropics, and often exposed to drought and heat stresses, with severe losses in production and deterioration in peanut quality worldwide (Nigam et al., 2005; Hamidou et al., 2012; Hamidou et al., 2013). About 90% of the world’s peanuts are cultivated in tropical and semi-arid regions, and ∼65% of United States peanuts are grown in dryland, and rainfed conditions (Hamidou et al., 2013). While peanuts tolerate early drought stress, it is more sensitive to drought and heat stress toward the reproductive phase. A temperature range between 25°C and 30°C is optimum for peanut growth and productivity. Temperature above 32°C negatively impacts yield and total biomass in peanuts (Cox, 1979; Golombek and Johansen, 1997; Prasad et al., 2003). Peanuts under drought stress are vulnerable to aflatoxin contamination due to infection caused by *Aspergillus flavus* (Hamidou et al., 2014), a toxic substance harmful to human and animal health, impacting the peanut trade internationally. Drought and heat stress also alters compositional changes in seed chemistry, including adverse effects on minerals (Dwivedi et al., 2013).

A meta-analysis involving over 120 published case studies of crop responses to combined drought and heat stress reveals that the combined effect significantly impacts yield by reducing harvest index, shortening the life cycle of crops, and altering seed number, size, and composition. Moreover, such impacts are more severe when the stress combination occurs during the crops reproductive phase (Cohen et al., 2021).

**FIGURE 1:**
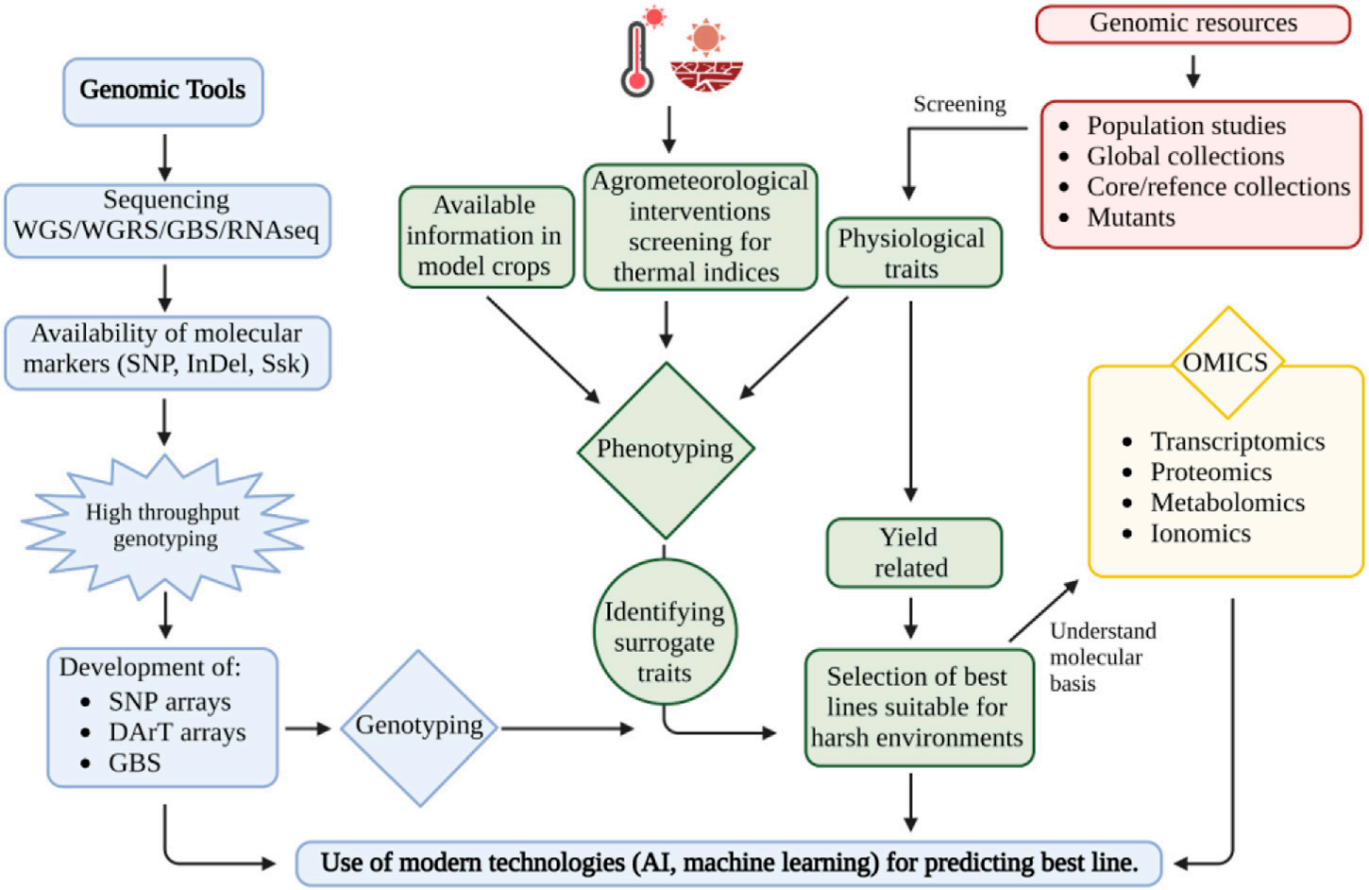
Utilization of genomic and genetic resources for developing peanuts for harsh environment conditions. This figure was created with BioRender.com.

Hence, understanding the physiological and molecular basis of drought and heat stress tolerance is the key to improving peanuts’ productivity in harsh environments (Figure 1). Here we provide synthesis to a wide range of plant responses to these stresses to harness variation toward developing stress-tolerant and productive peanut germplasm, which may be recycled in breeding programs or could be deployed in commercial production after assessing their performance in each production system.

## High-throughput phenomics to accelerate data collection in germplasm/breeding populations

### Drought stress

Traditional screening for drought tolerance refers to conditions in which the germplasm/breeding populations are exposed to varying moisture stress levels in field environments. While the control plots receive optimal irrigation throughout the crop cycle, in the stressed plots, water is withheld at a critical stage (i.e., reproductive phase) for a specific period and then released similar to control (irrigated) plots. The difference in pod yield between irrigated and drought-stressed plots is measured as a response to drought stress. The genotypes that show the least reduction in pod yield under stress are classified as tolerant to drought (Craufurd et al., 2003; Krishnamurthy et al., 2007; Kakani et al., 2015; Akbar et al., 2017; Abadya et al., 2021). This type of stress is categorized as a mid- or end-of-season drought. The occurrence, frequency, and intensity of stress in natural field environments are difficult to predict, i.e., the crop may face stress at any given time during the rainy season. This type of stress is defined as intermittent drought. In a situation like this, the genotypes/breeding populations are exposed to intermittent drought stress while the corresponding control plots receive optimal irrigation throughout the crop cycle. Genotypes with the least difference in pod yield between stressed and control (irrigated) plots are identified as tolerant to intermittent drought (Gangappa et al., 2006; Ratnakumar and Vadez, 2011; Hamidou et al., 2012; Vadez and Ratnakumar, 2016). Such screening methods are time- and resource-intensive, and subject to bias due to genotype-by-environment interaction effects. However, screening only for yield response under drought or high temperature does not give us information regarding the physiological and genetic mechanisms that may be involved in the observed yield under drought tolerance.

Water use efficiency (WUE) is a critical trait in breeding for drought- and heat-stress tolerance in peanuts. However, long-term transpiration is challenging to measure under field conditions. Technically, it requires using lysimeters, which is economically unfeasible for typical peanut breeding programs. Surrogates for WUE have been identified, including carbon isotope discrimination (Δ^13^C), specific leaf area (SLA), SPAD chlorophyll meter reading (SCMR), canopy temperature depression (CTD), normalized difference vegetation index (NDVI), and visual rating of leaf wilting (Wright et al., 1994; Ravi et al., 2011; Luis et al., 2016; Vadez and Ratnakumar, 2016). Still, the labor and time required to collect these measurements are prohibitive for large populations and in multiple environments. High-throughput phenotyping in plants is thus a significant bottleneck in breeding programs.

High-throughput plant phenotyping (HTPP) employs unmanned aerial vehicle (UAV), unmanned ground vehicle (UGV), robotics, various imaging technologies, and advanced data analytics to enable efficient and effective characterization of complex plant traits for screening germplasm or breeding populations. Most HTPP research has been focused on major row crops such as cotton, maize, soybean, and wheat, whereas HTPP research in peanuts only started in recent years. Table 1 summarized a list of existing HTPP studies where the predicted traits were or could be used to screen drought tolerance in peanuts.

**TABLE 1:** Exisiting high-throughput plant phenotyping studies related to drought tolerance screening in peanuts.

Sensor	Platform	Data analytics	Traits	References
RGB	UAV	VIs	leaf wilting rating	Balota and Oakes (2017)
		VIs + linear/ML regression	leaf area index, lateral growth	Sarkar et al. (2021)
		digital surface model	plant height	Sarkar et al. (2020)
	pushcart	CNN-based pod detection and counting	pod yield	Bidese et al. (2021)
	minirhizotron	CNN-based root semantic segmentation	root architecture	Xu et al. (2020)
multispectral	UAV	NDVI	disease rating, pod yield	Patrick et al. (2017), Chen et al. (2020)
hyperspectral	UAV	VI + ensemble ML	pod yield, pod count, biomass	Bagherian et al. (2022)
		VNIR reflectance + CNN		
thermal	handheld	canopy temperature	transpiration, pod yield	Balota and Oakes (2017)
LiDAR	UGV	point cloud analysis	plant height	Yuan et al. (2019)
GPR	pushcart	image thresholding	pod yield	Dobreva et al. (2021)

Vegetation index (VI), Machine learning (ML), convolutional neural network (CNN), unmanned aerial vehicle (UAV), unmanned ground vehicle (UGV), ground penetrating radar (GPR), light detection and ranging (LiDAR), visible near-infrared (VNIR).

Infrared thermal imaging of canopy temperature is currently the most accurate and direct method, as drought-induced stomatal closure causes a reduction in transpiration and, thus, a decrease in canopy temperature. Typically, a thermal camera would be mounted on a UAV along with other imaging sensors for high-throughput multi-modal imagery acquisition over a large area. Balota and Oakes (2017) first evaluated UAV-based red-green-blue (RGB) and near-infrared (NIR) imaging and handheld RGB and thermal imaging for HTPP of 26 peanut cultivars in a drought experiment. RGB color indices, NDVI, and CTD, were found to have strong to moderate correlations with visual leaf wilting rating, pod yield, sound mature kernel, and crop value at the end of water stress imposition. Aerial RGB color indices coupled with statistical learning models have been reported to achieve a 90% accuracy in predicting visual leaf wilting ratings (Sarkar et al., 2021). CTD can detect drought stress before visible leaf wilting occurs (Balota and Oakes, 2017). Other related HTPP studies in peanuts were focused on peanut canopy morphology. Although they may not provide early detection of drought and heat stress, peanut canopy architecture traits can potentially influence plant water use. Peanut canopy height has been quantified accurately by both Light Detection and Ranging (LiDAR) sensors on a high-clearance motorized cart (Yuan et al., 2019) and digital surface models derived from a UAV-based RGB imaging platform (Sarkar et al., 2020). In addition, leaf area index (LAI) and lateral growth can be predicted by training statistical and machine learning models on aerial vegetation indices (Sarkar et al., 2021). Deep roots can increase plant water uptake capability, contributing to drought and heat tolerance. Minirhizotron imaging has been used to infield HTPP of peanut root architecture, and the UNet-based semantic segmentation method has been effective and robust in detecting root pixels (Xu et al., 2020).

HTPP of other agronomic traits in peanuts also has been standardized to accelerate peanut breeding efforts. Pod yield has been the most important trait to measure in peanut breeding programs. UAV remote sensing-based vegetation indices of peanut canopy at critical phenological stages, such as the pod-filling stage, have shown their value for early yield prediction (Balota and Oakes, 2017; Jewan et al., 2022). For direct sensing of peanut pods, ground penetrating radar has shown the potential to explain yield variability up to 51% (Dobreva et al., 2021). In addition, HTPP of infield peanut pods after inversion presents a low-cost approach for pod yield prediction at the end of the growing season. Bidese et al. (2021) employed a push-cart system to collect top-viewing and side-viewing RGB videos of inverted peanut plants in the field. They explored Mask R-CNN-based peanut pod detection coupled with multivariate linear regression for pod yield prediction. The imaged scenes were highly complex, with heavy occlusions between peanut pods, leaves, and vines. The potential of this approach needs further investigation to account for pod size and variability in visibility. Disease incidence may become a confounding factor for screening of drought and heat tolerant peanut genotypes and affect subsequent data analysis and selection process. UAV multispectral imaging-derived vegetation indices accurately predict visual ratings of tomato spot wilt virus and bacterial wilt in peanuts (Patrick et al., 2017; Chen et al., 2020).

In addition to the studies reviewed above, other HTPP technologies can facilitate breeding drought and heat tolerance in peanuts. Hyperspectral imaging provides both high resolutions in spatial and spectral dimensions for phant phenotyping applications (Sarić et al., 2022). Compared to a typical multispectral camera with five wide spectral bands (blue, green, red, red edge, and near-infrared), a visible-near-infrared (VNIR) hyperspectral camera produces hundreds of narrow spectral bands between 400 and 1000 nm wavelengths, which can reveal a far more detailed spectral signature of plant organs. New normalized difference vegetation indices (i.e., FOSBNDI-1, FOSBNDI-2, and COSBNDI) derived from UAV-based hyperspectral data were effective at predicting maize leaf water content at the V6 stage in conjunction with a machine learning model (Raj et al., 2021). In contrast to engineering spectral features, all spectral bands can also be exploited by statistical or deep learning methods (i.e., partial least squares regression or deep convolutional neural networks) for maize leaf water content prediction with automatic feature selection or learning (Ge et al., 2016; Rehman et al., 2020). Similar approaches can help rapidly screen peanut genotypes with high water uptake capability.

Regarding remote sensing of peanut yield and yield components, Bagherian et al. (2022) recently evaluated predicting biomass, pod count, and pod yield using UAV-based hyperspectral imaging and machine learning techniques for single peanut plants of an F_1_ population in a mid-season drought experiment using rainout shelters. Eighteen days after the drought was found to result in the highest prediction accuracies for the three agronomic traits (*R* ^2^ = 0.52–0.61). On the other hand, high photosynthetic capacity can act as a mechanism for drought and heat tolerance. Currently, photosynthetic parameters such as the maximum carboxylation rate of Rubisco (*V*_*c,max*_), maximum electron transport rate (*J*_*1800*_), maximum electron transport rate supporting RuBP regeneration (*J*_max_), maximal light-saturated photosynthesis (*P*_max_), and chlorophyll content are often measured using a portable photosynthesis instrument (e.g., LI-COR LI-6800), which can be extremely time- and labor-intensive. Hyperspectral imaging and machine learning have been found effective in predicting photosynthetic parameters (Fu et al., 2019; Meacham-Hensold et al., 2020). This can be applied to track the temporal dynamics of photosynthetic activity in peanuts for drought experiments under rainout shelters and identify superior genotypes that quickly recover from drought stress. Chlorophyll fluorescence imaging provides a two-dimensional image instead of a point measurement using a chlorophyll fluorimeter. The resultant high spatial resolution can reveal spatial variability in photosynthetic performance on a single leaf or between leaves on a plant. Chlorophyll fluorescence imaging has been used to study drought and heat stress response in tomato, *Arabidopsis*, and wheat in controlled environments (Wang C. et al., 2018; Yao et al., 2018; Abdelhakim et al., 2021) and grain sorghum under field conditions (Herritt et al., 2020). Chlorophyll fluorescence measurements, such as maximum and operating quantum efficiencies of photosystem II photochemistry (*F*_*V*_ */F*_*M*_, *Φ*_PSII_ = (*F*_m_’–*F*)/*F*_m_’), can indicate plant drought and heat stress earlier than the occurrence of morphological changes such as leaf wilting. Table 2 lists some HTPP studies for drought and/or heat stress in crop species that could be applied to peanuts.

**TABLE 2:** HTPP methods that have not been studied for drought and heat stress phenotyping in peanuts. Shortwave infrared (SWIR).

Sensor	Platform	Data analytics	Traits	References
hyperspectral	UAV	VIs	leaf water content	Raj et al. (2021)
	pushcart	VNIR reflectance + PLSR	*V*_*c,max*_, *J*_*1800*_, *P*_max_, Chl *a*/*b* ratio, Chlorophyll content	Meacham-Hensold et al. (2020)
	handheld	VNIR + SWIR reflectance + Ensemble ML	*V*_*c,max*_, *J*_max_	Fu et al. (2019)
chlorophyll fluorescence imaging	field-based gantry	kinetic chlorophyll fluorescence curve	*F*_*V*_ */F*_*M*_	Herritt et al. (2020)
	controlled imaging chamber		*F*_*q*_ *’/F*_*m*_ *’, F*_*V*_ */F*_*M*_ *, Φ*_*PSII*_	Abdelhakim et al. (2021), Wang H. et al. (2018), Yao et al. (2018)

With the advancements in HTPP and increasing availability of high-dimensional sensor-based phenotypic datasets, phenomic-assisted selection has recently been proposed and evaluated. This analysis uses HTPP data instead of genomic data as input to the statistical models in genomic selection. The phenomic choice can achieve comparable predictive accuracy compared to genomic selection for crops such as wheat, soybean, and maize (Rincent et al., 2018; Parmley et al., 2019; Weiß et al., 2022). The advantages include low cost and robustness across different environments (Rincent et al., 2018). The phenomic selection performed more accurately for complex traits such as grain yield than traits controlled by a few genes (Zhu et al., 2022). Since drought and heat tolerance are considered complex traits, peanut breeders are expected to benefit from phenomic selection and reduce the labor and time required for screening diverse populations.

### Heat stress

A similar approach, like discarding of ultrasusceptible types to drought stress, is also recommended for screening for heat stress tolerance in peanut (Akbar et al., 2017; Table 3). Developing a reliable index and identifying traits for acquired thermotolerance in peanuts is necessary for breeding heat-tolerant varieties. Several reports observed the genotypic variability in peanut’s heat tolerance for partitioning dry matter to pods and kernels, fruit set, membrane stability, and chlorophyll fluorescence (Srinivasan et al., 1996; Vara Prasad et al., 2001; Craufurd et al., 2003). The detached leaf assay method was used to screen the sixteen genotypes from US minicore accessions along with standard checks were evaluated for acquired thermoterance. Here, the change in the temperature sensitivity of chlorophyll accumulation was used as an indicator of acquired thermotolerance. However, in this study, there was no significance effect of thermotolerance on seed weight was observed, hence it was difficult to relate chlorophyll content with heat tolerance (Selvaraj et al., 2011; Table 3). In another study, peanut seedlings of diverse genotypes for heat tolerance were screened using temperature induction response techniques. About 2 days old peanut seedlings were exposed to sublethal temperature from 28°C to 54°C for 5 h, followed by the lethal temperature at 54°C for 3 h. The expression patterns of stress-responsive genes were analyzed in selected heat-tolerant genotypes; genes related to HSP90, DREB2A, and *LEA4-2* were highly induced (Kokkanti et al., 2019) that can be used as markers for screening. Lipid peroxidation can cause severe membrane injury (MI) during abiotic stress. As such, it can be measured to assess the degree of stress in peanuts (Blum and Ebercon, 1981; Srinivasan et al., 1996; Bajji et al., 2002). Ribose, hydroxyproline, and saturated fatty acids were negatively correlated with MI, which can be used as stress tolerance parameters. Hence, there is a need to emphasize on the practical and robust screening methods to select for heat stress tolerance in peanut. Of late, studies have been carried out to utilize the thermal indices (growing degree days, phenothermal indices, heat use efficiency) for studying heat tolerance in peanut (Sukanth, 2022) and efforts are being made to map the heat tolerance related traits in groundnut (Sharma et al., unpublished).

**TABLE 3:** Genotypes exhibiting heat stress tolerance along with its responsive trait.

Genotype	Subspecies	Heat stress tolerance responsive/tolerant trait	References
COC038	*A. hypogaea*	Chlorophyll accumulation/HSP production	Selvaraj et al. (2011)
COC041	*A. fastigiata*	Chlorophyll accumulation/HSP production	Selvaraj et al. (2011)
COC050	*A. fastigiata*	Chlorophyll accumulation/HSP production	Selvaraj et al. (2011)
COC068	*A. fastigiata*	Chlorophyll accumulation/HSP production	Selvaraj et al. (2011)
ICGS 76	*A. hypogaea*	Chlorophyll accumulation/Acquired thermal tolerance	Selvaraj et al. (2011)
ICGS 44	*A. hypogaea*	High yield/HSP expression	Chakraborty et al. (2018)
ICG 8242	*A. hypogaea*	High Yield	Chakraborty et al. (2018)
796	*A. hypogaea*	Low relative injury and High Yield	Craufurd et al. (2003)
ICG 1236	*A. hypogaea*	Cardinal Temperature for pollen germination	Craufurd et al. (2003)
ICGV 86021	*A. hypogaea*	Crop growth rate, plant growth rate and partitioning	Craufurd et al. (2003)
ICGV 87281	*A. hypogaea*	Microsporogenesis, Flowering, Cellular membrane stability, Crop growth rate and Pod growth rate	Craufurd et al. (2003)
ICGV 92121	*A. hypogaea*	Microsporogenesis and Flowering	Craufurd et al. (2003)
SPT 06-07	*A. hypogaea*	Chlorophyll index, less membrane damage and pollen viability	Craufurd et al. (2003)
ICGV 97182	*A. hypogaea*	High stress tolerance index (STI) value	Akbar et al. (2017)
ICGV 01232	*A. hypogaea*	High STI value	Akbar et al. (2017)
ICGV 07013	*A. hypogaea*	High STI value	Akbar et al. (2017)
ICGV 07213	*A. hypogaea*	High STI value	Akbar et al. (2017)
ICGV 89280	*A. hypogaea*	High STI value	Akbar et al. (2017)
ICGV 00350	*A. hypogaea*	High STI value	Akbar et al. (2017)
ICGV 03057	*A. hypogaea*	High STI value	Akbar et al. (2017)
ICGV 06420	*A. hypogaea*	High STI value	Akbar et al. (2017)
ICGV 02266	*A. hypogaea*	High STI value	Akbar et al. (2017)
ICGV 03109	*A. hypogaea*	High STI value	Akbar et al. (2017)
ICGV 06099	*A. hypogaea*	High STI value and high kernel Fe- and Zn- content	Akbar et al. (2017)
ICGV 07273	*A. hypogaea*	High STI value	Akbar et al. (2017)
ICGV 00351	*A. hypogaea*	High STI value and drought-tolerant	Akbar et al. (2017)
ICGV 07268	*A. hypogaea*	High STI value	Akbar et al. (2017)
ICGV 06039	*A. hypogaea*	High STI value and Superior pod yield	Akbar et al. (2017)
ICGV 07148	*A. hypogaea*	High STI value	Akbar et al. (2017)
ICGV 03042	*A. hypogaea*	High STI value and Superior pod yield	Akbar et al. (2017)
ICGV 05032	*A. hypogaea*	High STI value	Akbar et al. (2017)
ICGV 07038	*A. hypogaea*	High STI value and Superior pod yield	Akbar et al. (2017)
ICGV 05155	*A. hypogaea*	High STI value	Akbar et al. (2017)
ICGV 06040	*A. hypogaea*	High STI value, Superior pod yield, and high kernel Fe- and Zn- content	Akbar et al. (2017)
ICGV 07012	*A. hypogaea*	High STI value and Superior pod yield	Akbar et al. (2017)
ICGV 06424	*A. hypogaea*	High STI value and Superior pod yield	Akbar et al. (2017)
ICGV 07246	*A. hypogaea*	High STI value and Superior pod yield	Akbar et al. (2017)
TG 37	*A. hypogaea*	High STI value	Akbar et al. (2017)
TAG 24	*A. hypogaea*	High STI value	Akbar et al. (2017)
ICG 4729	*A. fastigiata*	High yielding-High Temperature	Hamidou et al. (2013)
ICG 5236	*A. hypogaea*	High yielding-High Temperature	Hamidou et al. (2013)
ICG 12879	*A. hypogaea*	High yielding-High/moderate Temperature	Hamidou et al. (2013)
ICG 15042	*A. hypogaea*	High yielding-High Temperature	Hamidou et al. (2013)
ICG 862	*A. hypogaea*	High yielding-High/moderate Temperature	Hamidou et al. (2013)
ICG 1668	*A. hypogaea*	High yielding-High Temperature	Hamidou et al. (2013)
ICG 2925	*A. hypogaea*	High yielding-High Temperature	Hamidou et al. (2013)
ICG 8285	*A. hypogaea*	High yielding-High/moderate Temperature	Hamidou et al. (2013)
ICG 11219	*A. hypogaea*	High yielding-High Temperature	Hamidou et al. (2013)
Derived RILs from JL 24 × 55–437	*A. hypogaea*	Heat use efficiency, phenothermal indices	Sukanth (2022)

## Physiological basis of stress tolerance

### Drought stress

Peanut shows different water needs at different developmental stages. The water demand is the highest at mid-pod filling stage because the peanut canopy covers all the ground and maintains open the stomata to maintain high photosynthesis to fill the growing pods (Stansell and Pallas, 1985; Rowland et al., 2012a; Rowland et al., 2012b). Understanding the main effects of drought on plant growth and yield may unfold the physiological basis of drought tolerance.

**FIGURE 2:**
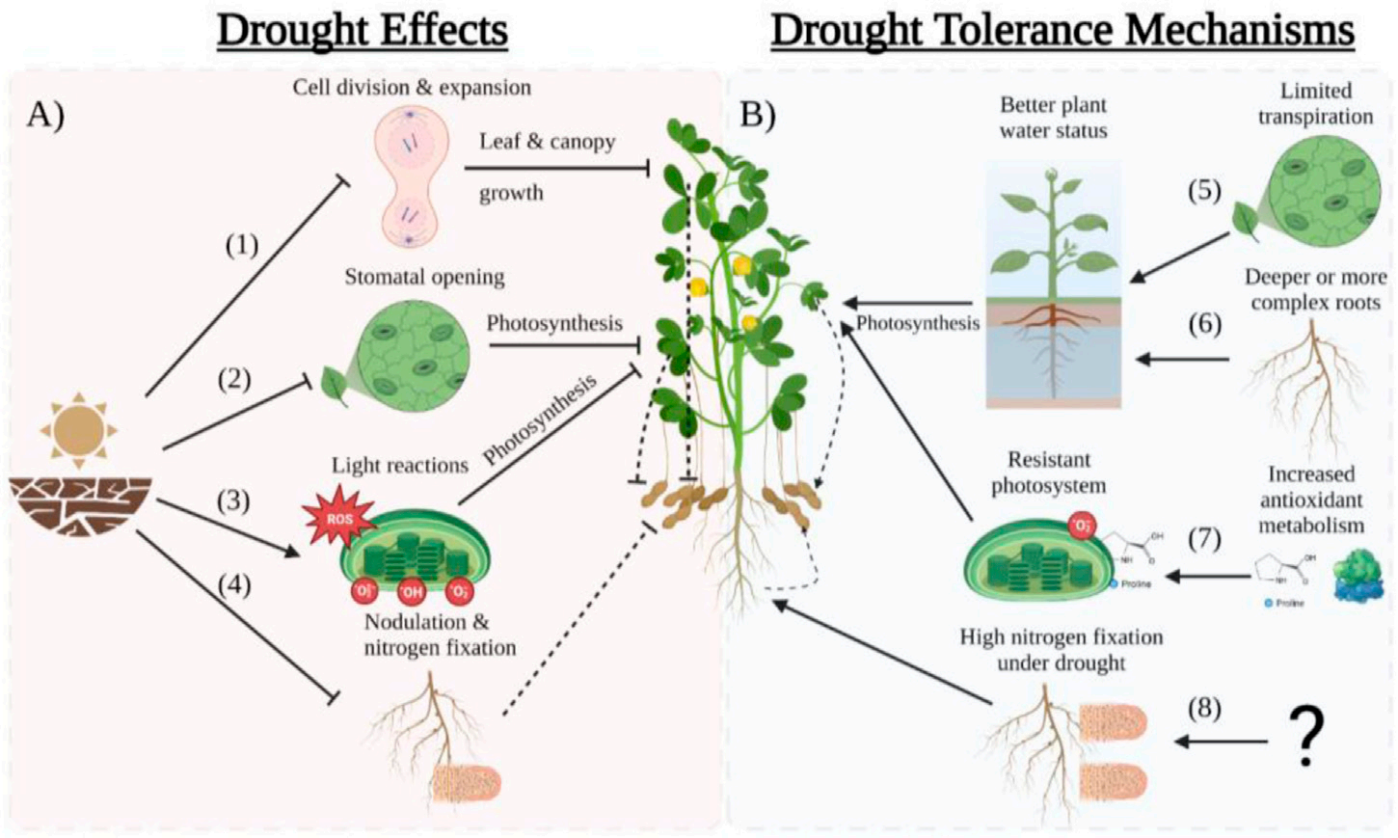
Scheme of drought effects **(A)** and tolerance mechanisms **(B)** in peanut. (1) Drought decreases the leaf water potential which inhibits cell division and expansion limiting leaf and canopy growth thus reducing pod yield. (2) The reduction of leaf water potential limits stomatal opening which reduces photosynthesis and therefore yield. (3) Drought produces reactive oxygen species (ROS) that damage the chloroplast membranes hindering the light reaction which result in decreased photosynthesis and yiled. (4) Drought inhibits nitrogen fixation in nodules by inhibiting the enzyme nitrogenase which reduces N availability resulting in lower yields. (5) Plants that reduces transpiration early in the drought period (water savers) are able to save water in the soil showing a better water status and therefore maintain moderate photosynthesis producing acceptable yields. (6) Plants with deeper or more dense root systems are able to extract more water to maintain good plant water status which allows the plant to photosynthetize more and maintain high yileds under drought. (7) Cultivars that produce more antioxidants such as proline are able to detoxify the ROS produced by drought maintaining a more healthy photosystems which results in higher photosynthesis and drought. (8) Cultivars that maintain high nitrogen fixation under drought are able to produce higher yields. However the underlying mechanisms of high nitrogen fixation under drought is unknown. This figure was created with BioRender.com.

The drying of the soil due to drought and the subsequent reduction in leaf water potential and cell turgor leads to the inhibition of cell division and elongation that results in slower leaf growth rates aimed at reducing transpiration at the canopy level (Figure 2) (Ribaut et al., 2009; Avramova et al., 2015). To preserve water in the soil and maintain an acceptable leaf water potential, peanuts tend to decrease stomatal conductance (*g*_*s*_) and transpiration resulting in reduced photosynthesis (Reddy et al., 2003; Pilon et al., 2018). Reduced leaf area expansion and lower photosynthesis per leaf area lead to a decline in canopy carbon assimilation that will reduce biomass accumulation and yield (Reddy et al., 2003). Plant traits that preserve soil moisture, such as high-water use efficiency (WUE) due to rapid stomatal closure, could increase drought tolerance (Devi et al., 2010; Devi and Sinclair, 2011; Shekoofa et al., 2015; Sinclair et al., 2017; Zhang et al., 2022). Contrarily, there are peanut cultivars that can maintain adequate plant water status and escape drought by collecting more water due to a more complex or deep root system (Rowland et al., 2012a; b; Zhang, et al., 2022). Utilization of genomic and genetic resources for developing peanuts for harsh environment conditions are illustrated in Figure 1.

When drought is maintained for long periods, and CO_2_ assimilation is reduced, the excess light not used for photosynthesis tends to produce reactive oxygen species (ROS) such as superoxide anion and hydrogen peroxide, H_2_O_2_ (Akcay et al., 2010; Laxa et al., 2019). ROS accumulation has been related to lipid peroxidation and thylakoid membrane damage (Lauriano et al., 2000; Quilambo, 2004). It decreases the effectiveness of Photosystems I and II (PSI and PSII) by producing non-stomatal limitations of drought that decrease photosynthetic efficiency resulting in reduced yield (Pilon et al., 2018). Peanut cultivars can tolerate these effects of drought by accumulating antioxidant substances that can reduce the accumulation of ROS to maintain higher photosynthetic rates during drought (Figure 2) (Akcay et al., 2010; Li et al., 2021).

Drought not only decreases plant growth and yield through a decrease in leaf and canopy photosynthesis (Reddy et al., 2003; Pilon et al., 2018) but also decreases nitrogen uptake by inhibiting the biological nitrogen fixation (BNF) in the roots (Pimratch et al., 2007). Nitrogen fixation is more sensitive to drought than photosynthesis because drought increases oxygen permeability to the nodule, inhibiting the activity of nitrogenase, the enzyme that catalyzes BNF (Parsons and Sunley, 2001). Drought also reduces the transport of the amino acid products of BNF from the nodule to the shoot, accumulates amino acids in the nodules, and creates a feedback mechanism that inhibits BNF (Peoples et al., 1986; Vessey et al., 2005). Thus, selecting genotypes with higher BNF under drought is another target to improve peanut yields (Sinclair et al., 1995; Sinclair, 2011; Devi et al., 2013). Peanut plants may therefore adapt diverse physiological attributes to balance productivity and stress tolerance as detailed herewith.

#### Limited Transpiration and High-Water Use Efficiency (WUE)

WUE (also referred to as transpiration efficiency, TE) is the amount of carbon assimilated as biomass per unit of water used by the crop (Medrano et al., 2015). When the atmosphere surrounding a plant gets drier, the plants tend to compensate by opening the stomata and increasing transpiration if they have enough water in the soil (Figure 2). This air drying can be simulated in a growth chamber by drying the air while measuring the plant transpiration by gravimetric methods (Devi et al., 2010). Under these conditions, some peanut cultivars can limit transpiration quicker than others when the environment gets drier by reducing their stomatal conductance, *g*_*s*_ (Devi et al., 2010; Devi and Sinclair, 2011; Shekoofa et al., 2015; Sinclair et al., 2017). This helps to save water in the soil that can “feed” the plant until maturity preserving biomass production and yield. This screening method has been used to select cultivars that later showed drought tolerance in field environments (Shekoofa et al., 2015). Reduced transpiration due to lower stomatal conductance maintains yield because of higher WUE. Vadez and Ratnakumar (2016) demonstrated that cultivars with high WUE can produce more yield under severe terminal drought conditions in a mini-lysimeter experiment under controlled field conditions. However, the high WUE trait may be a disadvantage under intermittent drought as the reduced stomatal conductance limits photosynthesis and biomass production compared with other crops that use more water (Blum, 2009; Polania et al., 2016). However, this yield penalty of high WUE cultivars have not been demonstrated until know and more research needs to be done in this area.

#### Effective Use of Water due to More Complex Root System

Effective use of water (EUW) refers to the amount of water that a plant can extract from the soil during the entire growing season and then use for transpiration, photosynthesis, biomass production and thus yield (Figure 2) (Blum, 2009). In common beans, cultivars with high EUW can maintain transpiration and photosynthesis for more time, resulting in higher yields under drought (Polania et al., 2016; Sanz-Saez et al., 2019). This mechanism has been detected and estimated by measuring the Δ^13^C of the biomass and selecting for high Δ^13^C in common beans (Farquhar et al., 1989; Polania et al., 2016; Sanz-Saez et al., 2019). Such genotypes use more water directly related to more profound or abundant root systems, as reported in common beans (White et al., 1990). Drought-tolerant peanut cultivars can exhibit high WUE or EUW. Peanut cultivars with high EUW do not show a yield advantage compared to high WUE under mid-season drought (Zhang et al., 2022). The high EUW capacity of these peanut cultivars has not yet been associated with a more profound or complex root system, as evidenced in the common bean (White et al., 1990). However, peanut cultivars with deeper or dense roots can extract more water to withstand drought (Songsri et al., 2008; Zhao et al., 2016). Zurweller et al. (2018) found that cultivars with more root development at deeper soil profiles (80 cm) do not result in drought tolerance in the mini-lysimeter environment. This conclusion may be affected by the fact that the roots are confined in a pot or only be relevant to the cultivars studied. For this reason, more research is needed to understand the role of different root morphological and anatomical characteristics on peanut drought tolerance.

#### Increased Antioxidant Metabolism to Reduce Adverse Effects of Reactive Oxygen Species (ROS)

Drought maintained for long periods can increase the production of ROS, which can damage proteins and lipids, ultimately reducing the efficiency of the photosynthetic system (Akcay et al., 2010; Laxa et al., 2019). To get protection from ROS, plants have evolved oxygen-scavenging systems consisting of non-enzyme antioxidant compounds such as proline, ascorbate, and glutathione and different antioxidant enzymes such as SOD, APX, CAT, POX, and GR (Bowler et al., 1992) (Figure 2). Drought-tolerant peanut cultivars showed high levels of CAT and APX that helped plants to decrease dangerous levels of H_2_O_2_. In contrast, high proline helped to maintain a higher osmotic potential to compensate for lower water potentials under drought (Akcay et al., 2010). In a more innovative approach, Banavath et al. (2018) produced a transgenic peanut line overexpressing a homeodomain-leucine zipper transcription factor (*AtHDG11*) which showed increased photosynthesis under drought conditions, probably due to more active antioxidant metabolism that reduces the ROS damage. In the U.S., the peanut industry does not encourage transgenic approaches as peanut is mostly used for human consumption, and transgenic food crops do not have high consumer approval. Thus, screening of diverse lines with high antioxidant activity is needed to find and introgress genotypes that are tolerant to drought and produce high levels of ROS (Mittler, 2002).

#### Maintaining High Biological N_2_-fixation (BNF) under Drought

Maintaining high BNF under drought has been documented as a tolerant trait for legumes (Figure 2). Using different physiological techniques, crop physiologists and breeders have been able to introgress this trait in soybean elite lines that resulted in commercial cultivars with high BNF and yield under drought (Sinclair, 2000; Chen et al., 2007; King et al., 2014). In peanuts, it has been also demonstrated that cultivars that maintain high BNF accumulate more biomass resulting in higher yields (Sinclair et al., 1995; Devi et al., 2013). There is a very little research in the literature that focuses on understanding the underlying mechanisms regulating nitrogen fixation under drought conditions for peanuts and therefore we do not know why these cultivars show high nitrogen fixation under drought. However there have been some new efforts to understand the regulation involved in the nodulation of peanuts (Peng et al., 2017, 2021). For example, there have been no reports in the literature in which a sizeable number of peanut cultivars have been screened for BNF and then introgressed in elite lines as in soybean (Sinclair, 2000; Chen et al., 2007; King et al., 2014). This is partly because determining BNF *in-situ* in the field is very difficult and costly. In soybean and common bean, tolerance of BNF to drought has been screened in diverse populations using the ^15^N natural abundance method to find new breeding lines (Steketee et al., 2019; Oladzad et al., 2020). With the discovery of non-nodulating peanut lines (Peng et al., 2021), using the ^15^N natural abundance method should facilitate the screening of diverse peanut populations under well-water and drought conditions to delineate genomic regions responsible for the maintenance of N_2_-fixation under drought. Such an approach could also result in the discovery of lines with high N_2_ fixation under well-water and drought conditions for use in introgression breeding programs in peanuts, as has been done in soybean.

#### Interaction between Drought Tolerant Traits

To our knowledge, no publications focus on understanding if there is any relationship between the drought, as mentioned earlier, tolerance mechanisms in peanuts. For BioRender obj example, cultivars with limited transpiration and high WUE, as they maintain a good water status in the plant, will probably show higher BNF as the plant is not suffering as much drought stress. This high BNF under drought is not a sign of direct tolerance caused by a more resistant nitrogenase activity to drought but the consequence of maintaining a better water status. Another example is the maintenance of a better antioxidant metabolism; the events that improved the antioxidant quality in transgenic plants also improved the water status of the plant by increasing WUE (Banavath et al., 2018). In this case, it is unclear if the overexpression of *AtHDG11* improves the antioxidant status of peanuts and then water status or *vice versa.* For these reasons, experiments that aim to separate between different drought-tolerance mechanisms would be important to identify parental lines that can introgress different drought-tolerant traits into breeding programs.

### Heat stress

**FIGURE 3:**
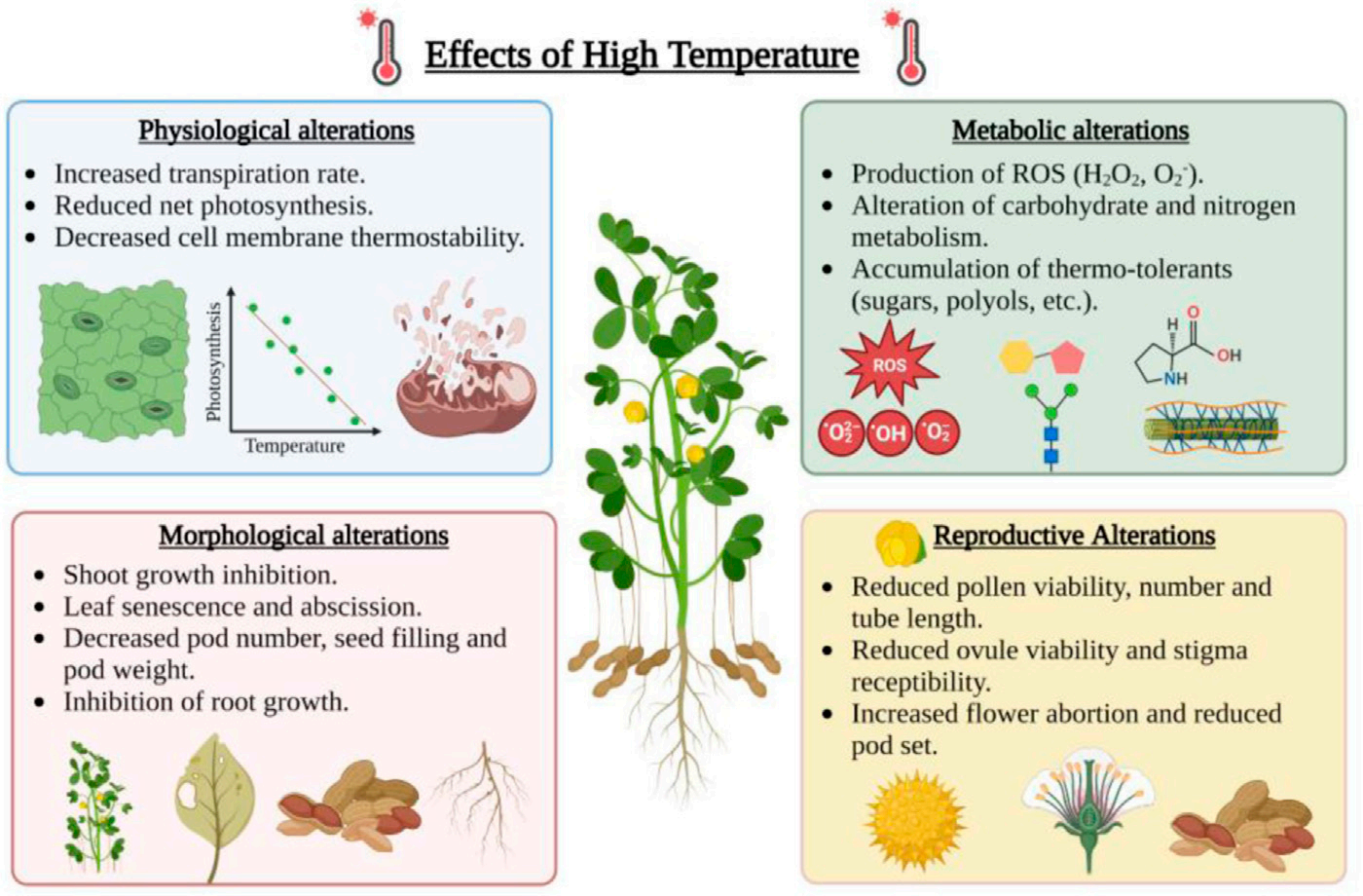
Effect of high temperature on physiological, metabolic, morphological and reproductive alterations in peanut. This figure was created with BioRender.com.

Higher temperatures can disrupt the physiological processes in plants, including a reduction in the rate of photosynthesis, degradation of chloroplast proteins, damage to PSII, lower relative water potential, ROS accumulation, and increase in lipid peroxidation (Crafts-Brandner and Salvucci, 2002; Dutta et al., 2009; Hasanuzzaman et al., 2020a). Heat stress affects the growth of male and female reproductive organs by impairing pollen tube growth, pollen viability, germination, egg viability, and fertilization (Figure 3). Microsporogenesis (3–6 days before flowering) and fruit set are two critical stages of peanut development which is affected by high temperatures (Craufurd et al., 2002, 2003). The late flowering to early seed setting stage was observed to be highly susceptible to high temperatures in peanuts (Prasad et al., 1999). However, the time of flower initiation at temperatures higher than 40°C/28°C day and night is the primary determinant of pod number in peanuts (Craufurd et al., 2000).

The impact of the elevated temperature is devastating as it affects membrane stability, inactivates chloroplast and mitochondrial enzyme function, causes protein degradation, reduces carbon metabolism, and alters cytoskeleton organization (Bita and Gerats, 2013) (Figure 3). Under heat stress, the thylakoid membrane and photosystem II (PSII) are severely damaged, disrupting the electron transport system and ATP synthesis during photosynthesis (Wang Q. L. et al., 2018). At temperatures higher than 38°C/32°C, the leaf chlorophyll content is reduced, which leads to less photosynthesis and low sugar content (Liu and Hang, 2000). Heat stress also affects the water status in the plant cells due to osmotic perturbation caused by low photosynthetic capacity, reduced sugar content, and higher transpiration rate (Hemantaranjan et al., 2018). High temperature under limited water conditions causes a reduction in relative water content and total water absorption rate, which contributes to total yield loss (Ashraf and Hafeez, 2004) (Figure 3). Stomatal conductance (*g*_*s*_) is directly correlated to the rate of photosynthesis and transpiration rate. During heat stress, stomatal density and stomatal conductance were found to be decreased in susceptible peanut varieties (Dash et al., 2020).

The efficient partitioning and allocation of carbon assimilate and photosynthates from source to sink are essential for plant growth and yield. Heat stress reduces carbon assimilation and partitioning and affects the plant source-to-sink relationship. Seed weight and yield depend on net photosynthesis and re-translocation of water-soluble carbohydrates from vegetative organs during seed filling (Fischer, 2011). The partitioning of dry matter allocation to shell relative to seed was higher in peanuts during higher temperatures. Higher temperature affects the photosynthates partitioning to the pod leading to low pod yield. The tolerant peanut genotypes were found to have higher crop growth rate and pod growth rate under heat-stress conditions than in the non-stress environment. Further, the photoperiod × temperature interaction influences the partitioning of dry matter into pods in peanuts (Nigam et al., 1994; Nigam et al., 1998; Akbar et al., 2017). Heat stress also damages the enzymes involved in nutrient metabolism and disrupts nutrient acquisition (Hungria and Kaschuk, 2014) (Figure 3).

Plants can tolerate the adverse effects of heat stress to some extent by modifying physiological and biochemical processes such as solute accumulation, osmotic adjustment, cellular homeostasis maintenance, and redox balance (Janská et al., 2010). One of the strategies for heat-stress tolerant cultivars is to have higher ceiling temperatures for pollen germination. Since heat stress significantly impacts peanut flowering, genotypes with a higher pollen germination ceiling temperature tend to be heat tolerant (Kakani et al., 2002; Chaudhary et al., 2022). Another effect of heat tolerance on peanuts or any other crop is membrane injury; increased unsaturated fatty acid levels caused by heat stress can disrupt membrane permeability, causing disrupted cellular homeostasis (Marcum, 1998). Photochemical changes during photosynthesis and reactive oxygen species (ROS) production due to heat stress can also affect membrane integrity (Bita and Gerats, 2013), causing membrane leakage. The heat-tolerant peanut varieties can be screened using an electrolyte leakage test or membrane injury test (MIT) by evaluating cell membrane thermostability (CMT) (Lauriano et al., 2000; Yeh and Lin, 2003; Akcay et al., 2010). Craufurd et al., 2002 reported the lower membrane injury in heat-tolerant peanut varieties. The higher heat tolerance is observed to be associated with higher gs value, photosynthesis rate, and stomatal conductance (Awasthi et al., 2014). The higher carotenoid content helps in preventing photo-oxidation of chlorophyll during stress. A study showed the heat tolerance peanut cultivars exhibited the higher carotenoid and higher chlorophyll level in the leaf (Dash et al., 2020). The protective mechanism of heat tolerance is associated with the activation of enzymatic and non-enzymatic ROS scavenging compounds. The higher activity of antioxidants such as SOD (Super oxide dismutase), CAT (Catalases), APX (Ascorbate peroxidase), and GR (Glutathione reductase) has been reported to provide plant thermostability (Kumar et al., 2013). Higher levels of total soluble sugar improve the thermotolerance of legume plants primarily by increasing water relations and gas exchange activities, thereby enhancing vegetative and reproductive growth (Ahmad et al., 2021a). Phytohormones such as abscisic acid (ABA) play a vital role in the stress tolerance of the plant by regulating its physiological processes. Plant growth regulators such as ABA, TU (Thiourea), GABA (Gamma-Amino butyric acid), and brassinosteroids help in enhancing the accumulation of soluble sugar, osmoprotectants, antioxidant enzymes, and gas exchange traits during heat stress tolerance (Ahmad et al., 2021b). Further, heat shock proteins (HSPs) play a crucial role in thermotolerance by maintaining protein structure and membrane integrity. Gene expression profile revealed that HSPs and heat shock factors (HSFs) are involved in tolerance during heat stress in legume crops (Zhang et al., 2015; Ma et al., 2016).

#### Genes/transcription factors that have targeted in model/crop plants for developing tolerant plant against heat/drought

Functional genomics and biotechnological techniques have been a valuable tools to identify and characterize genes associated with agronomic traits for the crop improvement. Differentially expressed genes related to storage proteins, fatty acid metabolism, oil production, biotic stress, *etc.*, have been identified and cloned using EST sequencing for the improvement of peanut variety (Jain et al., 2001; Chen et al., 2012). Candidate genes/QTLs for thermotolerance such as HSPs, compatible osomoprotectants, antioxidants, *etc.*, have been identified which can be used to develop heat tolerant crops using transgenic approach (Chaudhary et al., 2022). An *E. coli* gene encoding trehalose-6-phosphate synthase/phosphatase (TPSP) overexpressed in tomato induced the expression of HsfA1, HsfA2, and HsfB1which further upregulated the heat tolerant HSPs, i.e., Hsp17.8, ER-sHsp, and Mt-sHsp (Lyu et al., 2018). Further, the overexpression of CaHsp25.9 in Capsicum improved the thermotolerance and increased the proline and SOD content in transformed lines (Feng et al., 2019). Pea plant overexpressing the heat shock factor gene HsfA1d from A. thaliana showed the higher activity of proteins related to antioxidative pathways such as SOD and APX activity and lower H_2_O_2_ accumulation during heat stress which further enhanced the thermotolerance of plant (Shah et al., 2020). In another study, the transgenic tomato overexpressing the cAPX helped in increasing the tolerance against heat stress (Wang et al., 2006). ROS generation disturbs the cellular processes during the heat stress. The activation of HSPs/HSFs involved in lowering the ROS accumulation *via* ROS scavenging pathway. The heat shock transcription factors HsfA2 and HsfA4 act as a H_2_O_2_ sensor and involved in the regulation of genes associated with ROS mitigation (Scarpeci et al., 2008). Sakuma et al., 2006 reported that the overexpression of a constitutive active form of transcription factor DREB2A CA induces drought-, salt-responsive as well as HSPs-related genes in *Arabidopsis* and provides significant tolerance to heat and water stress.

## Sources of variation for drought and heat stress tolerance

### Cultivated genepool

Plant genetic resources are the basic raw materials to empower crop improvement programs. The peanut gene pool consists of cultigen (*Arachis hypogaea*) with its many landraces, improved cultivars, and more than 83 wild species of the genus *Arachis* (Gregory et al., 1980). Cultivated peanut is an allotetraploid (2n = 4× = 40) that originated from natural hybridization of two diploid species, *Arachis duranensis* (A-genome) and *Arachis ipaensis* (B-genome**)** followed by spontaneous polyploidization of the hybrid and its subsequent domestication by Neolithic proto-farmers 6-10,000 years ago (Bertioli et al., 2016). Cultivated peanut germplasm is classified into two main subspecies: *A. hypogaea* and *A. fastigiata*. The subsp. *hypogaea* contains two botanical varieties: *hypogaea* (Virginia type) and *hirsuta*, while subsp. *fastigiata* contains four botanical varieties: *fastigiata* (Valencia type), *peruviana*, *aequatoriana*, and *vulgaris* (Spanish type). All six botanical varieties have unique morphological characteristics that separate them from one another (Krapovickas et al., 1994). Worldwide over 15,000 peanut accessions are preserved in the national and international genebanks, including 1823 accessions in N.I. Vavilov Research Institute of Plant Industry, Russia; 14,320 in ICRISAT, India; 7,432 in USDA, Griffin; and 9103 in China (Pandey et al., 2012). Assessment of such a large gene pool for agronomically beneficial traits is economically not feasible and also subject to high genotype by environment interaction. Smaller subsets representing a diversity of the entire collection of given species preserved in a genebank are ideal resources to evaluate for agronomic and stress tolerance traits.

Reduced subsets in the form of core (Frankel, 1984) and mini core (Upadhyaya and Ortiz, 2001) collections provide smaller subsets of germplasm that could be used to mine valuable traits from entire germplasm collections more efficiently instead of screening germplasm as a whole collection. Several such sets are reported for efficient utilization of genetic resources in peanut breeding and genetics (Ding et al., 2022; Dwivedi et al., 2008; Holbrook and Stalker, 2003; Holbrook and Dong, 2005; Upadhyaya et al., 2002a and; 2002b). The U.S. peanut mini-core collection has been effectively used for the identification of interesting alleles and traits for breeding programs for traits related to drought tolerance (Wang H, et al., 2016; Wang M. L. et al., 2016; Zhang et al., 2019; Li et al., 2022; Patel et al., 2022; Zhang et al., 2022). End-of-season drought tolerance was reported in 15 accessions after evaluating ICRISAT peanut mini core collection and selected based on pod yield, SPAD, and SLA measurements (Upadhyaya, 2005).

Assessment of 150 peanut genotypes under rainout shelters showed significant differences in pod yield, relative water content (RWC), SLA, leaf dry matter content (LDMC), chlorophyll fluorescence (CHF), Δ^13^C, photosynthesis, and stomatal conductance (g_s_), and resulted in 13 accessions resistant to midseason drought stress. In addition, gas exchange parameters were measured regularly during the drought and recovery to monitor dynamic changes in photosynthesis and g_s_ under stress. Genotypes with high yield, Δ^13^C, photosynthesis, and g_s_ under stress were classified as water spenders while genotypes with equally high yields but with low Δ^13^C and g_s_ and moderate photosynthesis under drought stress were classified as water savers (Zhang et al., 2022). The previous reports on screening US peanut mini core collection across three irrigation treatments over 2 years and two field locations unfolded five accessions (PI 502120, PI 493329, Line 8, Georgia-06G, AU-NPL-17) as resistant to drought. These accessions had high SPAD, flowering, and paraheliotropism (Selvaraj et al., 2009; Belamkar, 2010). Paraheliotropism refers to condition in plants wherein the plants orient their leaves parallel to incoming rays of light. Elsewhere several germplasms tolerant to drought were reported, which showed significant differences in root depth, length, and density. The tolerant germplasms (#11, #34, #49, A596, Datangyou, Fenghua 1, Huayu 17, Huayu 21, Huayu 22, Huayu 25, Huayu 27, Ji 0212-4, Jihua 2, Jihua 4, L19, L121, L146, Luhua 14, NC6, Rugaoxiyangsheng, Shanhua 11, Tai 0125, Tai 0005, Taihua 4, Tangke 8, Xianghua 2008, Xianghua 55, Xuhua 13, Yuanza 9102, Yuanza 9307, Yueyou 7, Zhonghua 8) display increased root to shoot ratio and the enhanced root length and density, particularly in the deep soil, in comparison to those grown under normal growth conditions. The dragon-type (runner) peanuts, such as ‘A596’ and ‘Rugaoxiyangsheng,’ were more tolerant, followed by Virginia, Spanish, intermediate, and Valencia peanuts (Yang et al., 2019).

A screen of 247 ICRISAT accessions tested under water-deficit environments in Africa and India identified a few most adapted genotypes [ICG 5891, ICG 6057, ICG 9777] across moderate- and high-temperature stressed environments. It showed significant genotype × environment interaction (Hamidou et al., 2012). Field assessment of advanced breeding lines under irrigated conditions during the postrainy season in heat-stressed (air temperature 35°C and above during flowering) and non-stressed (air temperature below 35°C irrigated) environments unfolded large variability for pod yield and physiological traits among genotypes in a heat stress environment. A pod yield reduction of 1.5%–43.2% was recorded under a heat-stressed climate. The genotypes under heat stress either yield poorly stable or increased pod yield under high-temperature stress. The heat-tolerant genotypes are identified based on the stress tolerance index. GJG 31, ICGV 87846, ICGV 03057, ICGV 07038, and GG 20 showed an increase in pod yield of 9.0%–47.0% at high temperatures, with a 0.65%–3.6% increase in pod growth rate. ICGV 06420, ICGV 87128, ICGV 97182, TCGS 1043, and ICGV 03042 were stable for pod yield and recorded a 0.25%–3.1% increase in pod growth rate. Thus, pod yield, hundred-seed weight, and pod growth rate under heat stress can be used to select heat-stress tolerant genotypes. Based on stress tolerance indices and pod yield performance, ICGVs 07246, 07012, 06039, 06040, 03042, 07038, and 06424 were identified as heat-tolerant genotypes and are used as parents in breeding programs in India (Akbar et al., 2017).

Developing reliable indices and traits for acquired thermotolerance in peanuts is necessary for breeding heat-tolerant varieties. Several reports observed the genotypic variability in peanut’s heat tolerance for partitioning dry matter to pods and kernels, fruit set, membrane stability, and chlorophyll fluorescence (Srinivasan et al., 1996; Vara Prasad et al., 2001; Craufurd et al., 2002).

### Wild *Arachis* species

Wild *Arachis* species originated in South America, selected during evolution in a range of environments and biotic stresses, which provided opportunities for the evolution of a rich source of allele diversity for resistance to several pests, including fungal diseases and drought tolerance (Bertioli et al., 2021). Thoppurathu et al. (2022) conducted A transcriptome analysis for *A. duranensis* (drought tolerant) and *Arachis stenosperma* (drought susceptible) revealed *A. duranensis* genotype had a higher number of transcripts related to DNA methylation or demethylation, phytohormone signal transduction and flavonoid production, transcription factors, and responses to ethylene, indicating that it is tolerant to drought stress. Exposing A and B genome diploids under progressive drying to examine curves of vapor pressure deficit (VPD) against a fraction of transpirable soil water (FTSW) revealed that *A. magna* and *A. duranensis* accessions had superior ability to regulate transpiration under water deficit stress (Leal-Bertioli et al., 2012).

In summary, although we have large number of accessions of peanut germplasm collection, a very limited numbers have been identified as abiotic stress tolerant genotypes. Some of those tolerant genotypes have been used in breeding programs, and others are available for further investigation as shown in Table 4.

**TABLE 4:** Summary of available abiotic stress tolerant germplasm in cultivated peanuts.

Abiotic type	Tolerant germplam	Evaluated trait
Drought stress	PI502120, PI 493329, AU-NPL 17, TifNV-High O/L, Line-4, Line-8, Georgia 06, C76-16, AU16-28, AU18-35, SPT06-6, Tifrunner, and PI196635	Yield under stress, Δ^13^C, photosynthesis, and g_s_
	#11, #34, #49, A596, Datangyou, Fenghua 1, Huayu 17, Huayu 21, Huayu 22, Huayu 25, Huayu 27, Ji 0212-4, Jihua 2, Jihua 4, L19, L121, L146, Luhua 14, NC6, Rugaoxiyangsheng, Shanhua 11, Tai 0125, Tai 0005, Taihua 4, Tangke 8, Xianghua 2008, Xianghua 55, Xuhua 13, Yuanza 9102, Yuanza 9307, Yueyou 7, Zhonghua 8	root depth, length, and density
	ICG 5891, ICG 6057, ICG 9777	pod yield and physiological traits
Heat stress	ICGVs 07246, 07012, 06039, 06040, 03042, 07038, and 06424	pod yield, hundred-seed weight, and pod growth rate under heat stress

## Molecular basis of stress tolerance

Abiotic stress tolerance is a complex phenomenon involving several small effect genes and their interaction with the environment. Understanding the molecular mechanisms of stress perception and adaptive/tolerance responses by the plants is essential for engineering crop plants for stress tolerance (Aravind et al., 2022).

### Drought stress

#### Genomic approaches to study drought tolerance

With the availability of several sequencing platforms, it is possible to detect many sequence variations. The most abundant markers available are single nucleotide polymorphisms (SNPs) obtained by several genotyping platforms. SNP markers are extensively used to assess crops’ genetic diversity and trait mapping, For high resolution trait mapping, a high-density SNP genotyping array having uniform genome coverage is required. Large genome size and low genetic diversity in the cultivated gene pool of peanuts driven the development of SNP arrays for high throughput genotyping. The availability of the Axiom_*Arachis* array of highly informative genome-wide SNPs, 58,233 SNPs after sequencing 41 diverse genotypes, allows for the generation of high throughput genotyping data to capture genetic diversity, high-resolution trait mapping and improve breeding efficiency (Pandey et al., 2017). Similarly, another 48K SNP array called “Axiom Archis2” was developed in which 1,674 haplotype-based SNP markers were included from 21 re-sequenced peanut accessions (Clevenger et al., 2018).

58K “*Axiom_Arachis*” array based bi-parental QTL mapping detected sixteen major main-effect QTLs (10.0%–33.9% PVE) for traits associated with drought tolerance, wherein the significant QTLs were detected for haulm weight (20.1% PVE) and SCMR (22.4% PVE) (Pandey et al., 2020). This study was also successful in identifying important candidate genes underlying these QTL regions, such as those encoding *glycosyl hydrolases*, *malate dehydrogenases*, *microtubule-associated proteins*, *transcription factors* such as *MADS-box*, *basic helix-loop-helix* (*bHLH*), *NAM*, *ATAF*, and *CUC* (*NAC*), and *myeloblastosis* (*MYB*).

Earlier literature reported 52 main-effect QTLs (M-QTLs) for nine different traits under two water regimes, accounting for low <12% PVE (Faye et al., 2015), while another study detected 153 main effect QTLs and 25 epistatic QTLs with low to moderate phenotypic variance for drought tolerance traits (Gautami et al., 2012). As the QTLs obtained for drought tolerance (Table 5) were showing low to moderate PVE, the attempts were not made to introgress the QTL regions for breeding for drought tolerance in groundnut.

**TABLE 5:** QTL and marker-trait association studies carried out for drought and heat tolerance in peanut.

S. No.	Abiotic stress	Mapping population	Molecular markers	Traits studied	QTLs/MTAs identified	References
1	Drought stress	TAG 24 × ICGV 86031	SSR	• Transpiration	105 main effect QTLs with 3%–33% PVE (few major QTLs)	Ravi et al. (2011)
				• Transpiration Efficiency		
				• Specific Leaf Area		
				• SCMR		
				• Canopy conductance		
				• Carbon discrimination ratio		
				• Yield parameters		
2	Drought stress	ICGS 76 × CSMG 84-1	SSR	• transpiration efficiency	153 Main effect QTS (No major QTLs)	Gautami et al. (2012)
		ICGS 44 × ICGS 76		• transpiration		
				• SCMR		
				• shoot dry weight		
				• Yield parameters		
3	Drought stress	300 genotypes (ICRISAT References collection)	DArT	• 50 agronomic traits including drought related traits	GWAS 84 MTAs under well-watered (8.83%–88.90% PVE) and 68 MTAs under drought stress (8.24%–90.09% PVE)	Pandey et al. (2014)
4	Drought stress	TAG 24 × ICGV 86031	SSR	• Yield parameters	52 QTLs with <12 PVE%	Faye et al. (2015)
				• SCMR		
5	Drought stress	TAG 24 ICGV 86031	Axiom_Archis Array (SNP)	• Transpiration	19 Major Main-effect QTLs with 10%–33.0% PVE	Pandey et al. (2020)
				• Transpiration Efficiency		
				• Specific Leaf Area		
				• SCMR		
				• Canopy conductance		
				• Carbon discrimination ratio		
				• Water use efficiency		
				• Leaf area		
				• Yield parameters		
6	Drought stress	125 genotypes	GBS DArTseq (SNP)	• leaf area index	GWAS 20 significant MTA (6.6–20.8%PVE)	Shaibu et al. (2020)
				• canopy temperature		
				• SCMR		
				• NDVI		
7	Drought stress	453 genotypes	Axiom_Arachis array (SNP)	• Leaf Chlorophyll Content	GWAS	Zou et al. (2022)
					5 MTA	
8	Heat stress	Tamrun OL01 × BSS 56	SSR	• Yield parameters	Single marker analysis (SMA) on Bulks (8 MTAs with 9.19%–17.69% PVE)	Selvaraj et al. (2009)
				• Pod and kernel traits		
				• Oil content		
9	Heat stress	JL 24 × 55–437	Transposable elements (AhTE) and SNP (GBS)	• Yield parameters	SMA (39 marker-trait association with 2.19%–5.78% PVE)	Sukanth (2022)
				• Thermal indices		
				• Membrane injury indices		
				• Heat Use Efficiency		
				• Phenological parameters		

Various genome-wide association studies (GWAS) are noteworthy for detecting significant associations for yield-related traits under abiotic stress. GWAS analysis performed in a reference set of peanuts reported a total of 152 significant marker-trait associations (MTAs) for different traits under well-watered (WW) and drought stress (DS) conditions. Eighty-four significant MTAs were detected under WW, explaining 8.83%–88.90% phenotypic variance, and sixty-eight significant MTAs were detected under DS, accounting for 8.24%–90.09% phenotypic variance (Pandey et al., 2014). A study involving ICRISAT peanut mini core collection (Upadhyaya et al., 2002) and four physiological traits (leaf area index, canopy temperature, SCMR, NDVI) resulted in 20 significant MTAs for the target traits, with 20% PVE for SCMR (Shaibu et al., 2020), while another study involving 58K Axiom_Arachis array data on 453 peanut accessions reported seven significant MTAs on six chromosomes and SNP AX-176820297 on Araip. B05 was associated with leaf chlorophyll content across the seasons. The gene Arahy. SDG4EV was found to be related to leaf chlorophyll content (Zou et al., 2022). The chlorophyll content is a critical component affecting photosynthesis in plants and is associated with abiotic stress adaptation (Singh and Thakur, 2018).

#### Transcriptomic approaches to study drought tolerance

Transcription factors (TFs) play a major role in abiotic stress adaptation. The expression of certain TFs is regulated by hormonal signals that trigger the expression of several stress-responsive genes. During drought stress, the abscisic acid (ABA), ethylene, and salicylic acid signaling pathways are induced to produce osmoregulatory substances to maintain ROS homeostasis in the plant cells (Miller et al., 2010). The osmolytes and osmoprotectants play a key role in protecting the plant cells by scavenging free radicals. Mannitol, an osmoprotectant, plays an important role in scavenging hydroxyl radicals generated during abiotic stresses. Dehydration responsive element-binding (DREB) TFs enhance plant tolerance to abiotic stresses by specifically binding dehydration response element/C-repeat (DRE/CRT) cis-elements to control downstream gene expression (Liu et al., 1998). A study of the transcriptome of genotypes that show differential behavior during drought stress could provide insights into the molecular mechanisms of stress tolerance. A genome-wide transcriptome study in the peanut genotypes C76-16 (drought tolerant) and Valencia-C (drought sensitive) using RNA-sequencing have revealed the activation of key genes involved in ABA and sucrose metabolic pathways during moisture-stress conditions. The differentially expressed genes (DEGs) under water deficit conditions include *Gcn5-related N-acetyltransferase* (*GNAT*), *BON1- associated protein*, *the lateral organ boundary* (*LOB*), and the *late embryogenesis abundance* (*LEA*), *etc.*, that are involved in the synthesis of osmoprotectants, photosynthates, abscisic acid, secondary metabolites, *etc.* (Bhogireddy et al., 2020). Another comparative transcriptome analysis in two peanut cultivars- NH5 (tolerant) and FH18 (sensitive), under drought stress, has indicated DEGs involved in pathways of GSH-related glutathione metabolism, glycolysis, glyoxylic acid, dicarboxylic acid ester metabolism, ABA and SA signal-transduction, ROS-scavenging, proline metabolism, cell wall sclerosis-related, and cutin and wax metabolism (Jiang et al., 2021). In the combined omics approach, the transcriptome and proteome of *Arachis duranensis*, the “A genome proginator” of cultivated peanut, was studied in water deficit conditions (Carmo et al., 2018). This study showed the downregulation of expression of certain genes [*Cht2*, *MLP-34*, *heat shock proteins* (*HS70*, *HS17.3*), *DOT-1,* and *MatK*] in the stressed root tissues. The information can further be utilized using appropriate genomics or transgenic/genome editing approaches to improve cultivated peanuts for drought tolerance.

### Heat stress

#### Genomics approaches to study heat tolerance

A bulk segregant analysis using single marker analysis (SMA) on a mapping population (Tamrun OL01 × BSS 56) showed eight marker-trait associations with 9.19%–17.69% PVE (Selvaraj et al., 2009). Preliminary studies on single marker analysis using mapping population JL 24 × 55–437 have suggested that the traits like heat use efficiency, thermal indices, specific leaf area, membrane injury indices can be used as surrogate traits for selecting heat tolerant genotypes (Aravind, 2021; Sukanth, 2022). Of late, there are efforts to identify QTLs for high-temperature tolerance related traits (Sharma et al., unpublished).

#### Transcriptomic approaches to study heat tolerance

The membrane stability during stress determines the heat tolerance level. With their chaperon activity, the heat shock proteins (HSPs) help the cells to tolerate heat stress by protecting essential enzymes and nucleic acids from denaturation and misfolding due to high temperature (Jain, 2000). Rapid induction of small HSPs was found during high-temperature stress conditions in peanuts and imparts physiological adaptation to heat stress (Chakraborty et al., 2018). A genome-wide analysis of HSFs using the genomic sequences of wild peanut ancestors, *A. duranensis* and *A. ipaensis*, detected sixteen orthologous pairs of highly syntenic Hsfs, clustered into three groups, between AA and BB genomes. These HSFs were also shown to have fungal elicitor-responsive elements that showed differential expression in cultivated peanuts under abiotic stress and *Aspergillus flavus* infection (Wang et al., 2017). The reproductive parts of peanuts are most affected during heat stress. A lipidome study on peanut anthers revealed that phosphatidylcholine (PC), phosphatidylethanolamine (PE), and triacylglycerol (TAG) lipid species contributed towards more than 50% of total lipids both in ambient and heat stress conditions. A recent study involving another lipidome reports a decrease in unsaturated lipid species containing 18:3 fatty acid and downregulation of the fatty acid desaturase 3-2 gene (FAD3-2) in peanuts under high temperatures (Zoong Lwe et al., 2020). A similar report also indicated the possibility of utilizing the information on membrane lipid unsaturation as an indicator of heat tolerance in soybean and peanuts (Rustgi et al., 2021).

Genes related to HSP90, dehydration-responsive element binding -2A (DREB2A), and late embryogenesis abundant 4-2 (LEA4-2) were highly induced during heat stress in a few peanut genotypes (Kokkanti et al., 2019). Heat stress generates ROS, such as superoxide radicals and H_2_O_2,_ which results in oxidative damage to biomolecules, lipid peroxidation, and reduced activities of ROS-scavenging enzymes (Dat et al., 2000). ROS signaling is linked to the activation of heat shock factors (HSFs) and heat shock proteins (HSPs) (Driedonks et al., 2015). The transcript and biochemical analysis demonstrated the higher expression and activities of gene encoding ascorbate peroxidase (APX), superoxide dismutase (SOD), and glutathione reductase (GR), whereas catalase (CAT) expression declined during heat stress combined with salinity and drought stress in peanut. An increase in lipid peroxidation was also observed during heat stress in peanuts (Patel et al., 2022).

Studies on heat tolerance in peanut is limited to physiological aspects and there is need to look into the molecular basis of heat tolerance. There is need to identify molecular markers and candidate genes with high PVE, that can potential be used in genomics-assisted breeding for abiotic stress tolerance.

## Combining stress tolerance, productivity, and nutritional quality through plant breeding and biotechnological-assisted approaches

### Plant breeding and selection

Breeding for drought and heat stress tolerance is extremely challenging due to the complexity associated with various stress-adaptive mechanisms, uncertainty in the onset and intensity of stress, and large genotype × environment interactions. Conventional crossing and selection to improve drought and heat stress tolerance have been met with limited success. The focus on yield, lack of simple physiological traits as a measure of tolerance, and complex inheritance (polygenes with small effects) contributed to the low genetic gain in stress tolerance breeding. A better understanding of the physiological basis of stress tolerance contributes to identifying and manipulating traits associated with yield in water-deficit stressed field environments (reference needed). A systematic characterization of germplasm and breeding lines resulted in genetically diverse germplasm varying in response to drought and heat stress (Table 5). Such genetic stocks showed large variations for physiological traits such as SLA, chlorophyll content, amount of water transpired, WUE and harvest index in a drought-stressed environment. Both empirical (yield in the stressed environment) and trait-based selection (SLA, SCMR) have led to the development and release of drought-tolerant peanut cultivars in India and Australia (Rachaputi, 2003). The trait-based selection, however, did not show a consistent superiority over the empirical selection for drought tolerance (Nigam et al., 2005). Integrating physiological traits or their surrogates in the selection scheme would be advantageous in selecting segregants that utilize water more efficiently and partition photosynthates more effectively into economic yield. A few drought-tolerant cultivars of wheat bred by trait-based breeding without any yield penalty have been released in Australia where drought is intense and terminal (Rebetzke et al., 2002; Richards et al., 2002; Condon et al., 2004). A combination of trait-based selection in an early stage of breeding and yield assessment at a later stage of cultivar development in target environments is needed to select for abiotic stress adaptation and yield in peanuts. High yield potential and higher resistance are difficult to target together; therefore, to avoid the yield penalty, cultivars with high yield potential were bred to moderate levels of stress tolerance (Nigam et al., 1991). A few commercial heat-tolerant varieties have been released through conventional breeding, such as 55-437, 796, ICG 1236, ICGV 86021, ICGV 87281, and ICGV 92121 (Craufurd et al., 2003).

### Introgression breeding using Wild *Arachis* species and their derivatives

Crop wild relatives are the source of variation for stress tolerance and productivity traits. Advanced backcross populations originating from synthetic amphidiploid as donors for wild alleles detected several QTLs with positive effects on pod/seed size and adaptation traits in water-limited environments (Essandoh et al., 2022). Progenies derived from a cross between synthetic allotetraploid (*A. duranensis × A. batizocoi*) and cultivated peanut improved photosynthetic traits and yield under water-deficit stress (Dutra et al., 2018). A root transcriptomic study involving drought tolerant (*A. duranensis*) and susceptible (*Arachis stenosperma*) wild species unfolded 1465 differentially expressed genes (DEGs) under drought stress and 366 polymorphic SNPs among DEGs. Three SNPs differentiated the two species and may be useful for selecting drought-tolerant lines (Thoppurathu et al., 2022). In addition, advanced backcross populations involving several synthetics (ISATGR 121250, ISATGR 278-18, ISATGR 265-5, ISATGR 40) and peanut cultivars (ICGV 91114, ICGV 87846, TMV 2, Tifrunner) exhibited considerable variability for morpho-agronomic traits (Sharma, 2017). They could be a good resource for screening for abiotic stress adaptation and use in peanut breeding. Synthetics are tetraploid derivatives originating from a cross between two diploid *Arachis* species from secondary gene pool with different genomes (Mallikarjuna et al., 2012).

### Genomics-assisted breeding

Advances in peanut genomics unfolded several QTLs and markers (SSRs, SNPs) and candidate genes associated with drought tolerance surrogate traits in peanut. For example, NDVI effectively predicts biomass and yield, while CTD is associated with transpiration efficiency and carbon dioxide assimilation. These markers explained between 6.6% and 20.8% phenotypic variation, with most markers identified on the A subgenomes and respective homeologous chromosomes on the B subgenomes (Shaibu et al., 2020). Such markers, upon validation, may be deployed in marker-assisted breeding for drought tolerance in peanuts. A number of NAM- and MAGIC-based populations are being developed in peanuts which may provide useful genetic and genomic resources to study and implement genomic-assisted breeding for enhanced resistance to drought and heat stress in peanuts (Holbrook et al., 2013; Varshney, 2016; Gangurde et al., 2019). Efforts are underway to apply genomic selection (GS) for predicting the phenotypes by studying their genotypic architecture in multi-environment breeding trials (Pandey et al., 2020), but study of GS related to abiotic stress tolerance is not yet defined in peanut. Marker-assisted breeding has successfully introduced resistance to nematodes, rust and leaf spot and improved oil quality in peanuts (Varshney et al., 2014; Bera et al., 2018; Ballén-Taborda et al., 2022).

Ascorbate peroxidase (APX), an antioxidant enzyme, contributes to ROS scavenging by decreasing hydrogen peroxide (H_2_O_2_) under environmental stresses. A comprehensive GWAS unfolded 166 *AhAPX* genes in the peanut genome, grouped into 11 main clusters, and have roles in oxidoreductase activity, catalytic activity, cell junction, cellular response to stimulus and detoxification, biosynthesis of metabolites, and phenylpropanoid metabolism. *AhAPX4/7/17/77/82/86/130/133* and *AhAPX160* showed significantly higher expression in diverse tissues/organs, while *AhAPX4/17/19/55/59/82/101/102/137* and *AhAPX140* were significantly upregulated under drought and cold stress, and phytohormones treatments. Functional characterization and validation of the *AhAPX* and SNPs may accelerate breeding programs to develop stress-tolerant peanut cultivars (Raza et al., 2022).

US peanut researchers at Clemson University reported lipid metabolism traits associated with heat tolerance which could be useful in determining lipid biomarkers to develop climate-resilient varieties. A targeted effort is underway in the US to develop new heat-resilient peanut cultivars using a range of heat-sensitive to heat-tolerant varieties such as “Bailey,” “Georgia 12Y,” “Phillips,” “Sugg,” “Tifguard,” and “Wayne” and a breeding line SPT06-07 (Zoong Lwe et al., 2020). The development of molecular marker linkage maps and identification of markers and QTLs for target traits paved the way to develop efficient breeding methods to generate new, improved heat-tolerant peanut cultivars. The availability of peanut genome sequences and advanced genomics tools will aid in efficiently utilizing genetic resources toward a generation of sustainable crop yield.

The classical breeding methods employed to enhance drought and heat stress tolerance have had limited success. Advances in next-generation sequencing and phenomics, availability of genome sequences, and advances in bioinformatics and biotechnological tools may open new windows of opportunities to improve abiotic stress adaptation in food crops, including peanuts.

### Transgenes and CRISPR/Cas9 Genome editing

Transgene and genome editing methods make up the core of the genetic engineering tool kit. These technologies alter a genome to create modified cell lines, new cultivars possessing valuable traits, or learn novel information about cellular processes or development. A transgene is a unit of genetic material inserted permanently or transiently into a cell where it is expressed to confer a phenotype. Efforts have been made to transfer genes of interest into the peanut genome *via Agrobacterium*-mediated transformation or particle bombardment. However, the successful production of transgenic plants has been limited since only a few genotypes were found to be transformable with relatively high efficiency. In peanuts, many factors, including a lack of efficient protocols to regenerate whole plants (Sharma and Anjaiah, 2000; Sharma and Ortiz, 2000; Geng et al., 2012; Chu et al., 2013) and bacterial interactions with peanut cells (Gelvin, 2003) may restrict transformation success and regeneration *via Agrobacterium*. The recalcitrance nature of many peanut cultivars to *Agrobacterium*-mediated transformation and regeneration is a challenging bottleneck for future peanut-based technology development. Therefore, there is a pressing need to explore suitable genotype-independent transformation methods, such as *in planta* transformation, which may avoid time-consuming tissue culture and regeneration processes.

Nevertheless, the technology has successfully deployed to create transgenic events in peanuts with enhanced drought, salt, and aflatoxin tolerance. Transgenic peanuts containing *AtDREB1A* confer tolerance to drought. Assessment of these events under varying moisture stress regimes and vapour pressure deficits (VPDs) yielded up to 24% improvement in seed yield largely due to increased harvest index and higher seed filling, and displayed 20%–30% lower pod yield reduction than WT under drought stress (Bhatnagar-Mathur et al., 2014). Another study led by Qin et al. (2011) reported that regulating the expression of the *IPT* gene by a water-deficit inducible promoter (P_SARK_) performed much better, maintained higher photosynthetic rates and stomatal conductance (*g*_*s*_), produced significantly more biomass, and yield under reduced irrigation conditions in greenhouse and field environments. Transgenic peanut plants overexpressing the *AtAVP1* were tolerant to both drought and salt stress, produced high biomass, and maintained higher photosynthetic and transpiration rates under reduced irrigation and saline conditions in the greenhouse. Additionally, transgenic peanuts expressing the transcription factors *AtNAC2* and *MuNAC4* from *Arabidopsis* and *Macrotyloma* showed high tolerance to drought, salt, and moisture stress and high yield in stressed environments (Pandurangaiah et al., 2014; Patil et al., 2014). Similarly, the expression of the *mtlD* (mannitol-1- phosphate dehydrogenase) in transgenic peanut plants conferred drought tolerance (Bhauso et al., 2014a; Bhauso et al., 2014b; Patel et al., 2017), and the overexpression of *GmMYB3a* into transgenic peanut plants displayed better physiological parameters with improved drought tolerance (He et al., 2020).

Since stress tolerance is a multigenic trait involving different signaling cascades, developing transgenic lines with more tolerance traits by transferring more than one gene is needed (Venkatesh et al., 2018). Co-expression of multiple genes in transgenic plants has shown improved stress tolerance compared to transgenic plants with single-gene. Using modified MultiSite Gateway approach (Vemanna et al., 2013) to simultaneously stack *Alfin1*, *PgHSF4,* and *PDH45* genes driven by individual promoters and terminators into a single vector resulted in transgenic peanut plants with improved stress tolerance, higher growth, and productivity than WT plants under drought-stress conditions (Table 6). Another successful example was that two-antifungal plant defensins *MsDef1* and *MtDef4.2* and two host-induced gene silencing of *aflM* and *aflP* genes were cloned into four binary vectors. These vectors were mobilized into *Agrobacterium*, resulting in transgenic peanuts with a near-immunity of aflatoxin contamination (Sharma et al., 2018). This gives hope that day is not far off to stacking genes cascade with suitable promoters for developing peanuts that combine aflatoxin resistance, tolerate drought as well productive. Conventional breeding has had limited success to achieving resistance to aflatoxin because of multiple mechanisms (*in-vitro* seed colonization, pre-harvest aflatoxin contamination and aflatoxin production) controlling aflatoxin contamination, phenotyping bottlenecks to measure different components of resistance, large genotype × environment interactions, and issues associated with pre- and post-harvest management of peanuts (Pandey et al., 2019).

**TABLE 6:** Genes used in transgenic peanut for tolerance of heat and drought stresses.

Gene	Function	Trait	References
*IPT*	Cytokinin biosynthesis	Drought	Li et al. (2013)
*AtDREB1A*	Transcription factor	Drought	Bhatnagar-Mathur et al. (2014); Sarkar et al. (2014)
*AtDREB2A, AtHB7, AtABF3*	Improve cellular tolerance	Drought and salt	Pruthvi et al. (2014)
*Alfin1, PgHSF, PDH45*	Stress-responsive transcription factor	Drought and oxidative stress	Ramu et al. (2016)
*AtHDG11*	Developmental regulator	Drought and salt	Banavath et al. (2018)
*MuNAC4*	Induce lateral root growth	Drought	Pandurangaiah et al. (2014), Venkatesh et al. (2022)

Overcoming tradeoffs is a significant breeding challenge when combining stress tolerance and crops productivity as many genes of minor effects are involved. Identifying gene variants with diverse functions to overcome tradeoffs should receive a greater investment of time and resources to balance crop growth, stress tolerance and productivity (Dwivedi et al., 2021).

Genome editing involves transgenes or occasionally only proteins with or without an RNA, which can modify existing genetic material in a targeted manner, creating insertions, deletions, or base modifications. This technology has provided an alternative approach to plant breeding and has been efficient in producing new cultivars and genetic resources within a relatively short period. The clustered regularly interspaced short palindromic repeats (CRISPRs) and CRISPR-associated protein (Cas) type II systems provide methods for rapidly and efficiently editing plant genomes. This is accomplished through an RNA (CRISPR) guided nuclease (Cas) induced targeted double-strand DNA break, which can be repaired through several pathways that may lead to mutation. Furthermore, genomic DNA can be modified by tethering various enzymes to a nuclease-deficient Cas protein, which may also introduce targeted mutations (Xie et al., 2015; Samanta et al., 2016; Li et al., 2019). Specifically, CRISPR/Cas9-related technologies have tremendously impacted functional genomics by enabling selective and specific alteration of genomic DNA sequences *in vivo* (Li et al., 2019; Scheben et al., 2017; Zhang et al., 2019). Genome editing has various applications in plants, including basic and applied biological research for developing advanced biotechnology products (Zhang et al., 2019) through forward and reverse genetics, targeted gene insertion, promoter modification, and splice variant generation. This is a valuable technique for functional gene analysis or trait alteration, and its effectiveness has been demonstrated in many plant species.

Moreover, gain-of-function mutations through this same approach have been reported in plants (Wang et al., 2022). Additionally, identifying other type II Cas proteins, such as Cas12a (Cpf1), is another important avenue in genome editing research as it opens additional genomic regions to modification due to alternative PAM: TTTV (V=C, G, A) utilization. The recent development of Cas protein variants fused to a variety of enzymes has also widened the application of CRISPR/Cas technology from inducing indels, gene insertion, and gene modification through targeted double-strand DNA breaks to produce targeted single base changes through a system known as base editing (BE) or manipulating the gene expression through the promoter region.

Although CRISPR/Cas tools have been successful in a wide variety of plant species, their application in peanuts is currently limited. Realizing the full potential of CRISPR/Cas-based genome editing in peanuts will require the development of a toolbox of validated CRISPR/Cas constructs and protocols for their utilization. Several research programs have been focused on establishing these systems in peanuts by developing gene editing and base editing technology to include vectors using CRISPR/Cas9 and CRISPR/Cas12a variants to induce indels, make DNA alterations through base editing, or regulate gene expression. Work has progressed to develop delivery methods and validate construct functionality. For example, we have developed several constructs for genome editing using the peanut *FAD2* genes as proof-of-concept experiments. Two CRISPR/Cas9 constructs, pDW3872, and pDW3877, have induced indels in *FAD2* with an efficiency of up to 32% and 24%, respectively (unpublished). Two base editing constructs, pDW3873, and pDW3876, were developed using nCas9 fused to the cytosine deaminases APOEBEC1 or PmCDA1, respectively. These constructs successfully induced C to T changes with an overall efficiency of up to 21% and 42%, respectively (unpublished). Additional constructs (pDW3882 and pDW3886) expressing the enzymes AsCpf1 or LbCpf1 were investigated for their editing efficiency. The latter was more effective in peanuts than the former (unpublished). These preliminary results demonstrate that genome editing using CRISPR/Cas systems is feasible in peanuts.

Overall, using transgenes and genome editing technology in peanuts comes down to developing genotype-independent transformation protocols, identifying genes of interest and proof of concept using suitable genome editing constructs. Application of these advances will greatly accelerate genetic improvement in peanuts leading to the efficient generation of new lines with desirable traits, which will benefit peanut producers, industry, and consumers. On the other hand, the presence of foreign genes in transgenic plants triggers biosafety regulations. However, a comparison of transgenic Golden rice GR2E and conventional rice showed no statistically significant differences in the concentrations of phytic acid or the levels of trypsin inhibitor and no differences in pest and disease reactions between them (Mallikarjuna Swamy et al., 2019; Mallikarjuna Swamy et al., 2021). Comparative assessment of transgenic wheat containing the sunflower gene, *HaHB4* conferring drought tolerance and improved yield in driest environments, is nutritionally equivalent to non-transgenic wheat lines (González et al., 2019; Miranda et al., 2022). Because CRISPR/Cas9-based genome editing plants can be transgene-free by crossing edited plant offspring, they are assigned a non-regulated status (Ahmad et al., 2021), such as the common button mushroom modified by the CRISPR system obtained a non-regulated status in 2016 (Waltz, 2016). ‘Sanatech Seed’ has launched the world’s first genome-edited high-GABA tomato with enhanced nutritional benefits for consumption in Japan. This tomato contains high levels of gamma-aminobutyric acid (GABA), an amino acid believed to aid relaxation and help lower blood pressure (https://www.isaaa.org/kc/cropbiotechupdate/article/default.asp?ID=18668).

### Concluding remarks

The evidence to date and predictions suggest the overall negative effect of climate change on agricultural production especially when more food, nutritious and safe, is required to feed the growing word population. In addition, the food and feed produced today is less nutritious, and faces an increased risk to contamination by mycotoxin producing fungi due to climate change.

Peanut at reproductive stage is ultra-susceptible to drought and heat stress, causing substantial loss to production and nutritional quality. The drought-stressed peanut is prone to aflatoxin contamination. Thus, aflatoxin contaminated peanut is hazardous to human and animal health, in addition to adversely impacting peanut trade.

Novel sources of resistance to drought in peanut gene pool has led researchers thoroughly investigate the physiological and molecular basis of stress tolerance, while the genes and markers associated with stress tolerance detected significant marker trait associations, which after validation may be deployed in genomics-assisted breeding in peanut. Greater resources are needed to unfold the genetic and molecular basis of heat stress tolerance as this trait in the past received less attention compared to drought research in peanut. A few reports indicate the feasibility of gene transfer by transgenic technology, with some events showing no growth-defense tradeoff, suggesting the transgene(s), a valid technology, to rapidly integrate stress tolerance gene(s) without yield penalty. Advances in developing peanut-based construct and their editing efficiency demonstrate that genome editing using CRISPR/Cas system is feasible in peanut. Public perception about the use of genetically modified and/or gene edited crops is gradually changing in favor of for food and feed uses and also for commercial cultivation, as the evidence to date suggests no significant changes in proximate composition between genetically engineered and conventionally-bred produce, except for the trait introduced.

## References

[B1] Abadya S. Shimelis H. Pasupuleti J. Mashilo J. Chaudhari S. Manohar S. S. (2021). Assessment of genetic diversity of groundnut (*Arachis hypogaea* L.) for kernel yield, oil and fodder quantity and quality under drought conditions. Crop Sci. 61, 1926–1943. 10.1002/csc2.20483

[B2] Abdelhakim L. O. A. Rosenqvist E. Wollenweber B. Spyroglou I. Ottosen C. O. Panzarová K. (2021). Investigating combined drought-and heat stress effects in wheat under controlled conditions by dynamic image-based phenotyping. Agronomy 11, 364. 10.3390/agronomy11020364

[B3] Ahmad A. Munawar N. Khan Z. Qusmani A. T. Khan S. H. Jamil A. (2021). An outlook on global regulatory landscape for genome-edited crops. Int. J. Mol. Sci. 22, 11753. 10.3390/ijms222111753 34769204 PMC8583973

[B4] Ahmad M. Waraich E. A. Zulfiqar U. Ullah A. Farooq M. (2021a). Thiourea application improves heat tolerance in camelina (Camelina sativa L. Crantz) by modulating gas exchange, antioxidant defense and osmoprotection. Ind. Crops Prod. 170, 113826. 10.1016/j.indcrop.2021.113826

[B5] Ahmad M. Waraich E. A. Tanveer A. Anwar-ul-Haq M. (2021b). Foliar applied thiourea improved physiological traits and yield of camelina and canola under normal and heat stress conditions. J. Soil Sci. Plant Nutr. 21, 1666–1678. 10.1007/s42729-021-00470-8

[B6] Akbar A. Manohar S. S. Variat M. T. Kurapati S. Pasupuleti J. (2017). Efficient partitioning of assimilates in stress-tolerant groundnut genotypes under high temperature stress. Agronomy 7, 30. 10.3390/agronomy7020030

[B7] Akcay U. C. Ercan O. Kavas M. Yildiz L. Yilmaz C. Oktem H. A. (2010). Drought-induced oxidativ damage and antioxidant responses in peanut (*Arachis hypogaea* L.) seedlings. Plant Growth Regul. 1, 21–28. 10.1007/s10725-010-9445-1

[B8] Araus J. L. Slafer G. A. Reynolds M. P. Royo C. (2002). Plant breeding and drought in C3 cereals: What should we breed for? Ann. Bot. 89 Spec No (7), 925–940. 10.1093/aob/mcf049(12102518 PMC4233799

[B9] Aravind B. Nayak S. N. Choudhary R. S. Gandhadmath S. S. Prasad P. V. V. Pandey M. K. (2022). “Integration of genomics approaches in abiotic stress tolerance in groundnut (*Arachis hypogaea* L.): An overview,” in Genomic designing of abiotic stress resistant oilseed crops. Editor Kole C. (Cham, Switzerland: Springer international), 149–197. ISBN: 9783030900434.

[B10] Aravind B. (2021). Molec.ular elucidation of heat stress in terms of physiological and biochemical parameters in groundnut (*Arachis hypogaea* L.). M.Sc Thesis. Dharwad: University of Agricultural Sciences.

[B11] Ashraf M. Hafeez M. (2004). Thermo-tolerance of pearl millet and maize at early growth stages: Growth and nutrient relations. Biol. Plant. 48, 81–86. 10.1023/b:biop.0000024279.44013.61

[B12] Avramova V. Abdelgawad H. Zhang Z. Fotschki B. Casadevall R. Vergauwen L. (2015). Drought induces distinct growth response, protection, and recovery mechanisms in the maize leaf growth zone. Plant Physiol. 169, 1382–1396. 10.1104/pp.15.00276 26297138 PMC4587441

[B13] Awasthi R. Kaushal N. Vadez V. Turner N. C. Berger J. Siddique K. H. M. (2014). Individual and combined effects of transient drought and heat stress on carbon assimilation and seed filling in chickpea. Funct. Plant Biol. 41, 1148–1167. 10.1071/FP13340 32481065

[B14] Bagherian K. Bidese R. Bao Y. Zhang Q. Sanz-Saez A. Chen C. (2022). “Phenotyping agronomic traits of peanuts using UAV-based hyperspectral imaging and deep learning,” in 2022 ASABE Annual International Virtual Meeting (American Society of Agricultural and Biological Engineers), 1. 10.13031/aim.202200814

[B15] Bajji M. Kinet J. M. Lutts S. (2002). The use of the electrolyte leakage method for assessing cell membrane stability as a water stress tolerance test in durum wheat. Plant Growth Regul. 36, 61–70. 10.1023/A:1014732714549

[B16] Ballén-Taborda C. Chu Y. Ozias-Akins P. Holbrook C. C. Timper P. Jackson S. A. (2022). Development and genetic characterization of peanut advanced backcross lines that incorporate root-knot nematode resistance from *Arachis stenosperma* . Front. Plant Sci. 12, 785358. 10.3389/fpls.2021.785358 35111175 PMC8801422

[B17] Balota M. Oakes J. (2017). “UAV remote sensing for phenotyping drought tolerance in peanuts,” in Autonomous air and ground sensing systems for agricultural optimization and phenotyping II, 10218, 81–87. SPIE. 10.1117/12.2262496

[B18] Banavath J. N. Chakradhar T. Pandit V. Konduru S. Guduru K. K. Akila C. S. (2018). Stress inducible overexpression of *AtHDG11* leads to improved drought and salt stress tolerance in peanut (*Arachis hypogaea* L.). Front. Chem. 6, 34. 10.3389/fchem.2018.00034 29552555 PMC5840212

[B19] Belamkar V. (2010). A first insight into population structure, linkage disequilibrium, and association mapping of drought tolerance-related traits in the U.S. peanut mini core collection. M.S. thesis. Lubbock, TX: Texas Tech University.

[B20] Bera S. K. Kamdar J. H. Kasundra S. V. Dash P. Maurya A. K. Jasani M. D. (2018). Improving oil quality by altering levels of fatty acids through marker-assisted selection of *ahfad2* alleles in peanut (*Arachis hypogaea* L.). Euphytica 214, 162. 10.1007/s10681-018-2241-0

[B21] Bertioli D. J. Cannon S. B. Froenicke L. Huang G. Farmer A. D. Cannon E. K. (2016). The genome sequences of *Arachis duranensis* and *Arachis ipaensis*, the diploid ancestors of cultivated peanut. Nat. Genet. 48 (4), 438–446. 10.1038/ng.3517 26901068

[B22] Bertioli D. J. Clevenger J. Godoy I. J. Stalker H. T. Wood S. Santos J. F. (2021). Legacy genetics of Arachis cardenasii in the peanut crop shows the profound benefits of international seed exchange. Proc. Natl. Acad. Sci. U. S. A. 118, e2104899118. 10.1073/pnas.2104899118 34518223 PMC8463892

[B23] Bhatnagar-Mathur P. Rao J. S. Vadez V. Dumbala S. R. Rathore A. Yamaguchi-Shinozaki K. (2014). Transgenic peanut overexpressing the DREB1A transcription factor has higher yields under drought stress. Mol. Breed. 33, 327–340. 10.1007/s11032-013-9952-7

[B24] Bhauso T. Radhakrishnan T. Kumar A. Mishra G. Dobaria J. Patet K. (2014a). Over-expression of bacterial *mtlD* gene in peanut improves drought tolerance through accumulation of mannitol. Sci. World J. 125967, 125967. 10.1155/2014/125967 PMC4243595 25436223

[B25] Bhauso T. D. Thankappan R. Kumar A. Mishra G. P. Dobaria J. R. Venkat Rajam M. (2014b). Over-expression of bacterial *mtlD* gene confers enhanced tolerance to salt-stress and water-deficit stress in transgenic peanut (*Arachis hypogaea*) through accumulation of mannitol. Aust. J. Crop Sci. 8, 413. 10.3389/fpls.2016.00935

[B26] Bhogireddy S. Xavier A. Garg V. Layland N. Arias R. Payton P. (2020). Genome-wide transcriptome and physiological analyses provide new insights into peanut drought response mechanisms. Sci. Rep. 10, 4071. 10.1038/s41598-020-60187-z 32139708 PMC7058030

[B27] Bidese R. Bao Y. Sanz-Saez A. Chen C. (2021). “Infield peanut pod counting using deep neural networks for yield estimation,” in 2021 ASABE Annual International Virtual Meeting. (American Society of Agricultural and Biological Engineers). 10.13031/aim.202101080

[B28] Bita C. E. Gerats T. (2013). Plant tolerance to high temperature in a changing environment: Scientific fundamentals and production of heat stress-tolerant crops. Front. Plant Sci. 4, 273. 10.3389/fpls.2013.00273 23914193 PMC3728475

[B29] Blum A. Ebercon A. (1981), Cell membrane stability as a measure of drought and heat tolerance in Wheat1. Crop Sci., 21: 43–47. 10.2135/cropsci1981.0011183X002100010013x

[B30] Blum A. (2009). Effective use of water (EUW) and not water-use efficiency (WUE) is the target of crop yield improvement under drought stress. Field Crops Res. 112, 119–123. 10.1016/j.fcr.2009.03.009

[B31] Bowler C. Montagu M. V. Inze D. (1992). Superoxide dismutase and stress tolerance. Annu. Rev. Plant Physiol. Plant Mol. Biol. 43, 83–116. 10.1146/annurev.pp.43.060192.000503

[B32] Boyer J. S. (1982). Plant productivity and environment. Science 218 (4571), 443–448. 10.1126/science.218.4571.443 17808529

[B33] Carmo L. S. T. Martins A. C. Q. Martins C. C. C. Passos M. A. S. Silva L. P. (2018). Comparative proteomics and gene expression analysis in *Arachis duranensis* reveal stress response proteins associated to drought tolerance. J. Proteomics 192, 299–310. 10.1016/j.jprot.2018.09.011, 30267876

[B34] Chakraborty K. Bishi S. K. Singh A. L. Zala P. V. Mahatma M. K. Kalariya K. A. (2018). Rapid induction of small heat shock proteins improves physiological adaptation to high temperature stress in peanut. J. Agron. Crop Sci. 204, 285–297. 10.1111/jac.12260

[B35] Chaudhary S. Devi P. HanumanthaRao B. Jha U. C. Sharma K. D. Prasad P. V. V. (2022) Physiological and molecular approaches for developing thermotolerance in vegetable crops: A growth, yield and sustenance perspective. Front. Plant Sci. 13:878498. 10.3389/fpls.2022.878498 35837452 PMC9274134

[B239] Chen N. Yang Q. Su M. Pan L. Chi X. Chen M. (2012). Cloning of six ERF family transcription factor genes from peanut and analysis of their expression during abiotic stress. Plant molecular biology reporter 30, 1415–1425.

[B36] Chen P. Sneller C. Purcell L. Sinclair T. King C. A. Ishibashi T. (2007). Registration of soybean germplasm lines R01-416F and R01-581F for improved yield and nitrogen fixation under drought stress. J. Plant Reg. 1, 166–167. 10.3198/JPR2007.01.0046CRG

[B37] Chen T. Yang W. Zhang H. Zhu B. Zeng R. Wang X. (2020). Early detection of bacterial wilt in peanut plants through leaf-level hyperspectral and unmanned aerial vehicle data. Comp. Electron. Agric. 177, 105708. 10.1016/j.compag.2020.105708

[B38] Chu Y. Bhattacharya A. Wu C. Knoll J. E. Ozias-Akins P. (2013). Improvement of peanut (*Arachis hypogaea* L.) transformation efficiency and determination of transgene copy number by relative quantitative real-time PCR. Vitro Cell. Dev. Biol. Plant 49, 266–275. 10.1007/s11627-013-9518-8

[B39] Clevenger J. P. Korani W. Ozias-Akins P. Jackson S. (2018). Haplotype-based genotyping in polyploids. Front. Plant Sci. 9, 564. 10.3389/fpls.2018.00564 29755500 PMC5932196

[B40] Cohen I. Zandalinas S. I. Huck C. Fritschi F. B. Mittler R. (2021). Meta-analysis of drought and heat stress combination impact on crop yield and yield components. Physiol. Plant. 171, 66–76. 10.1111/ppl.13203 32880977

[B41] Condon A. G. Richards R. A. Rebetzke G. J. Farquhar G. D. (2004). Breeding for high water use efficiency. J. Exp. Bot. 55, 2447–2460. 10.1093/jxb/erh277 15475373

[B42] Cox F. R. (1979). Effect of temperature treatment on peanut vegetative and fruit growth 1. Peanut Sci. 6, 14–17. 10.3146/i0095-3679-6-1-4

[B43] Crafts-Brandner S. J. Salvucci M. E. (2002). Sensitivity of photosynthesis in a C4 plant, maize, to heat stress. Plant Physiol. 129, 1773–1780. 10.1104/pp.002170 12177490 PMC166765

[B44] Craufurd P. Q. Wheeler T. R. Ellis R. H. Summerfield R. J. Prasad V. P. V. (2000). Escape and tolerance to high temperature at flowering in groundnut (*Arachis hypogaea* L.). J. Agric. Sci. 135, 371–378. 10.1017/S0021859699008394

[B45] Craufurd P. Q. Prasad P. V. V. Summerfield R. J. (2002). Dry matter production and rate of change of harvest index at high temperature in peanut. Crop Sci. 42, 146–151. 10.2135/cropsci2002.1460 11756265

[B46] Craufurd P. Q. Prasad P. V. Kakani G. V. Wheeler T. R. Nigam S. N. (2003). Heat tolerance in groundnut. Field crop. Res. 80, 63–77. 10.1016/s0378-4290(02)00155-7

[B47] Dash D. Vp C. Bo K. (2020). Impact of heat stress on physiological and yield components under varied temperature regimes in groundnut cultivars. J. Pharmacogn. Phytochemistry 9, 1060–1066.

[B48] Dat J. Vandenabeele S. Vranová E. Van Montagu M. Inze D. Van Breusegem F. (2000). Dual action of the active oxygen species during plant stress responses. CMLS, Cell. Mol. Life Sci. 57, 779–795. 10.1007/s000180050041 10892343 PMC11147059

[B49] Devi M. J. Sinclair T. R. (2011). Diversity in drought traits among commercial southeastern US peanut cultivars. Int. J. Agron. 2011, 1–7. 10.1155/2011/754658

[B50] Devi J. M. Sinclair T. R. Vadez V. (2010). Genotypic variation in peanut for transpiration response to vapor pressure deficit. Crop Sci. 50, 191–196. 10.2135/cropsci2009.04.0220

[B51] Devi J. M. Rowland D. L. Payton P. Faircloth W. Sinclair T. R. (2013). Nitrogen fixation tolerance to soil water deficit among commercial cultivars and breeding lines of peanut. Field Crops Res. 149, 127–132. 10.1016/j.fcr.2013.04.026

[B52] Ding Y. Qiu X. Luo H. Huang L. Guo J. Yu B. (2022). Comprehensive evaluation of Chinese peanut mini-mini core collection and QTL mapping for aflatoxin resistance. BMC Plant Biol. 22, 207. 10.1186/s12870-022-03582-0 35448951 PMC9027753

[B53] Dobreva I. D. Ruiz-Guzman H. A. Barrios-Perez I. Adams T. Teare B. L. Payton P. (2021). Thresholding analysis and feature extraction from 3D ground penetrating radar data for noninvasive assessment of peanut yield. Remote Sens. 13, 1896. 10.3390/rs13101896

[B54] Driedonks N. Xu J. Peters J. L. Park S. Rieu I. (2015). Multi-level interactions between heat shock factors, heat shock proteins, and the redox system regulate acclimation to heat. Front. Plant Sci. 6, 999. 10.3389/fpls.2015.00999 26635827 PMC4647109

[B55] Dutra W. F. Guerra Y. L. Ramos J. P. C. Fernandes P. D. Silva C. R. C. Bertioli D. J. (2018). Introgression of wild alleles into the tetraploid peanut crop to improve water use efficiency, earliness and yield. PLOS ONE 13, e0198776. 10.1371/journal.pone.0198776 29889864 PMC5995397

[B56] Dutta S. Mohanty S. Tripathy B. C. (2009). Role of temperature stress on chloroplast biogenesis and protein import in pea. Plant Physiol. 150, 1050–1061. 10.1104/pp.109.137265 19403728 PMC2689951

[B57] Dwivedi L. Puppala N. Upadhyaya H. D. Manivannan N. Singh S. (2008). Developing a core collection of peanut pecific to Valencia market type. Crop Sci. 48, 625‒632.

[B58] Dwivedi S. Sahrawat K. Upadhyaya H. Ortiz R. (2013). Food, nutrition and agrobiodiversity under global climate change. Adv. Agron. 120, 1‒128. 10.1016/B978-0-12-407686-0.00001

[B59] Dwivedi S. L. Reynolds M. P. Ortiz R. (2021). Mitigating tradeoffs in plant breeding. iScience 24, 102965. 10.1016/j.isci.2021.102965 34466788 PMC8384922

[B60] Essandoh D. A. Odong T. Okello D. K. Fonceka D. Nguepjop J. Sambou A. (2022). Quantitative trait analysis shows the potential for alleles from the wild species*Arachis batizocoi* and *A. duranensis* to improve groundnut disease resistance and yield in east Africa. Agronomy 12, 2202. 10.3390/agronomy12092202

[B61] Farquhar G. D. Ehleringer J. R. Hubick K. T. (1989). Carbon isotope discrimination and photosynthesis. Annu. Rev. Plant Physiol. Plant Mol. Biol. 40, 40503–40537. 10.1146/annurev.pp.40.060189.002443

[B62] Faye I. Pandey M. K. Hamidou F. Rathore A. Ndoye O. Vadez V. (2015). Identification of quantitative trait loci for yield and yield related traits in groundnut (*Arachis hypogaea* L.) under different water regimes in Niger and Senegal. Euphytica 206, 631–647. 10.1007/s10681-015-1472-6 26594055 PMC4643859

[B63] Feng X. H. Zhang H. X. Ali M. Gai W. X. Cheng G. X. Yu Q. (2019). A small heat shock protein CaHsp25. 9 positively regulates heat, salt, and drought stress tolerance in pepper (Capsicum annuum L.). Plant Physiol. biochem. 142, 151–162. 10.1016/j.plaphy.2019.07.001 31284139

[B64] Fischer R. A. (2011). Wheat physiology: A review of recent developments. Crop Pasture Sci. 62, 95–114. 10.1071/cp10344

[B66] Frankel O. H. (1984). “Genetic perspectives of germplasm conservation,” in Genetic manipulation: Impact on man and society. Editors Arber W. K. Llimensee K. Peacock W. J. Starlinger P. (Cambridge: Cambridge Univ. Press), 161–170.

[B67] Fu P. Meacham-Hensold K. Guan K. Bernacchi C. J. (2019). Hyperspectral leaf reflectance as proxy for photosynthetic capacities: An ensemble approach based on multiple machine learning algorithms. Front. Plant Sci. 10, 730. 10.3389/fpls.2019.00730 31214235 PMC6556518

[B68] Gangappa E. Ravi N. Veerakumar G. N. (2006). Evaluation of groundnut (*Arachis hypogaea* L.) genotypes for temperature tolerance based on temperature induction response (TIR) technique. Indian J. Genet. Plant Breed. 66, 127–130.

[B69] Gangurde S. S. Wang H. Yaduru S. Pandey M. K. Fountain J. C. Chu Y. (2019). Nested association mapping (NAM)-based genetic dissection uncovers candidate genes for seed and pod weights in peanut (*Arachis hypogaea*). Plant Biotechnol. J. 18, 1457–1471. 10.1111/pbi.13311 31808273 PMC7206994

[B70] Gautami B. Pandey M. K. Vadez V. Nigam S. N. Ratnakar P. Krishnamurthy L. (2012). Quantitative trait locus analysis and construction of consensus genetic map for drought folerance traits based on three recombinant inbred line populations in cultivated groundnut (*Arachis hypogaea* L.). Mol. Breed. 30, 757–772. 10.1007/s11032-011-9660-0 22924017 PMC3410028

[B71] Ge Y. Bai G. Stoerger V. Schnable J. C. (2016). Temporal dynamics of maize plant growth, water use, and leaf water content using automated high throughput RGB and hyperspectral imaging. Comp. Electron. Agric. 127, 625–632. 10.1016/j.compag.2016.07.028

[B72] Gelvin S. B. (2003). Agrobacterium-mediated plant transformation: The biology behind the “gene-jockeying” tool. Microbiol. Mol. Biol. Rev. 67, 16–37. 10.1128/mmbr.67.1.16-37.2003 12626681 PMC150518

[B73] Geng L. Niu L. Gresshoff P. M. Shu C. Song F. Huang D. (2012). Efficient production of Agrobacterium rhizogenes-transformed roots and composite plants in peanut (*Arachis hypogaea* L.). Plant Cell Tiss. Organ Cult. 109, 491–500. 10.1007/s11240-012-0113-1

[B74] Golombek S. D. Johansen C. (1997). Effect of soil temperature on vegetative and reproductive growth and development in three Spanish genotypes of peanut (*Arachis hypogaea l.*). Peanut Sci. 24, 67–72. 10.3146/i0095-3679-24-2-1

[B75] González F. G. Capella M. Ribichich K. F. Curín F. Giacomelli J. I. Ayala F. (2019). Field-grown transgenic wheat expressing the sunflower gene *HaHB4* significantly outyields the wild type. J. Exp. Bot. 70, 1669–1681. 10.1093/jxb/erz037 30726944 PMC6411379

[B76] Gregory W. C. Krapovickas A. Gregory M. P. (1980). “Structure, variation, evolution, and classification in Arachis,” in Structure, variation, evolution, and classification in Arachis, 469–481.

[B77] Hamidou F. Ratnakumar P. Halilou O. Mponda O. Kapewa T. Monyo E. (2012). Selection of intermittent drought stress tolerant lines across years and locations in the reference collection of groundnut (*Arachis hypogaea* L.). Field Crops Res. 126, 189–199. 10.1016/j.fcr.2011.10.009

[B78] Hamidou F. Halilou O. Vadez V. (2013). Assessment of groundnut under combined heat and drought stress. J. Agron. Crop Sci. 199, 1–11. 10.1111/j.1439-037X.2012.00518.x

[B79] Hamidou F. Rathore A. Waliyar F. Vadez V. (2014). Although drought intensity increases aflatoxin contamination, drought tolerance does not lead to less aflatoxin contamination. Field Crops Res. 156, 103–110. 10.1016/j.fcr.2013.10.019

[B80] Hasanuzzaman M. Bhuyan M. H. M. Zulfiqar F. Raza A. Mohsin S. M. Mahmud J. A. (2020a). Reactive oxygen species and antioxidant defense in plants under abiotic stress: Revisiting the crucial role of a universal defense regulator. Antioxidants 9, 681. 10.3390/antiox9080681 32751256 PMC7465626

[B81] He Y. Mu S. He Z. Wang B. Li Y. (2020). Ectopic expression of MYB repressor *GmMYB3a* improves drought tolerance and productivity of transgenic peanuts (*Arachis hypogaea* L.) under conditions of water deficit. Trans. Res. 29, 563–574. 10.1007/s11248-020-00220-z 33161505

[B82] Hemantaranjan A. Malik C. P. Bhanu A. N. (2018). Physiology of heat stress and tolerance mechanisms-an overview. J. Plant Sci. Res. 34, 51–64. 10.32381/jpsr.2018.34.01.7

[B83] Herritt M. T. Pauli D. Mockler T. C. Thompson A. L. (2020). Chlorophyll fluorescence imaging captures photochemical efficiency of grain sorghum (*Sorghum bicolor*) in a field setting. Plant Methods 16, 109. 10.1186/s13007-020-00650-0 32793296 PMC7419188

[B84] Holbrook C. C. Dong W. (2005). Development and evaluation of a mini core collection for the U. S. peanut germplasm collection. Crop Sci. 45, 1540–1544. 10.2135/cropsci2004.0368

[B85] Holbrook C. C. Stalker H. T. (2003). Peanut breeding and genetic resources. Plant Breed. Rev. 22, 297–356. 10.1002/9780470650202.ch6

[B86] Holbrook C. C. Isleib T. G. Ozias-Akins P. Chu Y. Knapp S. J. Tillman B. (2013). Development and phenotyping of recombinant inbred line (RIL) populations for peanut (*Arachis hypogaea* L.). Peanut Sci. 40, 89–94. 10.3146/ps13-5.1

[B87] Hungria M. Kaschuk G. (2014). Regulation of N_2_ fixation and NO3−/NH4+ assimilation in nodulated and N-fertilized *Phaseolus vulgaris* L. exposed to high temperature stress. Environ. Exp. Bot. 98, 32–39. 10.1016/j.envexpbot.2013.10.010

[B88] IPCC (2022). “Climate change (2022): Impacts, adaptation, and vulnerability,” in Contribution of working group II to the sixth assessment report of the intergovernmental panel on climate change. Editors Pörtner H.-O. Roberts D. C. Tignor M. Poloczanska E. S. Mintenbeck K. Alegría A. (Cambridge, UK and New York, NY, USA: Cambridge University Press), 3056. 10.1017/9781009325844

[B89] Jain A. K. Basha S. M. andHolbrook C. C. (2001). Identification of drought responsive transcripts in peanut(*Arachishypogaea* L.). Mol. Biotechnol. Genet. 4, 2. 10.2225/vol4-issue2-fulltext-2

[B90] Jain V. K. (2000). Fundamental of plant physiology. New Delhi xS. Chand Publishing. ISBN: 8121904625.

[B91] Janská A. Marsík P. Zelenková S. Ovesná J. Janska A. (2010). Cold stress and acclimation - what is important for metabolic adjustment? Plant Biol. (Stuttg) 12 (3), 395–405. 10.1111/j.1438-8677.2009.00299.x 20522175

[B92] Jewan S. Y. Y. Pagay V. Billa L. Tyerman S. D. Gautam D. Sparkes D. (2022). The feasibility of using a low-cost near-infrared, sensitive, consumer-grade digital camera mounted on a commercial UAV to assess Bambara groundnut yield. Int. J. Remote Sens. 43, 393–423. 10.1080/01431161.2021.1974116

[B93] Jiang C. Li X. Zou J. Ren J. Jin C. Zhang H. (2021). Comparative transcriptome analysis of genes involved in the drought stress response of two peanut (*Arachis hypogaea* L.) varieties. BMC Plant Biol. 21, 64. 10.1186/s12870-020-02761-1 33504328 PMC7839228

[B94] Kakani V. G. Prasad P. V. V. Craufurd P. Q. Wheeler T. R. (2002). Response of*in vitro* pollen germination and pollen tube growth of groundnut (*Arachis hypogaea* L.) genotypes to temperature. Plant, Cell Environ. 25, 1651–1661. 10.1046/j.1365-3040.2002.00943.x

[B95] Kakani V. G. Timothy R. Wheeler P. Craufurd Q, Rao C. N. R. (2015). “Effect of high temperature and water stress on groundnuts under field conditions. combined stresses in forests,” in Combined stresses in plants. Physiological, molecular and biochemical aspects. Editor Mahalingham R. (Springer), 159–180. 10.1007/978-3-319-07899-1_8.2015

[B96] King Z. Serrano J. Roger Boerma H. Li H. (2014). Non-toxic and efficient DNA extractions for soybean leaf and seed chips for high-throughput and large-scale genotyping. Biotechnol. Lett. 36, 1875–1879. 10.1007/s10529-014-1548-8 24863292

[B97] Kokkanti R. R. Hindu V. Latha P. Vasanthi R. P. Sudhakar P. Usha R. (2019). Assessment of genetic variability and molecular characterization of heat stress tolerant genes in *Arachis hypogaea* L. through qRT-PCR. Biocatal. Agric. Biotechnol. 20, 101242. 10.1016/j.bcab.2019.101242

[B98] Krapovickas A. Gregory W. C. Williams D. E. Simpson C. E. (1994). Taxonomia del genero *Arachis* (*Leguminoae*). Bonplandia VIII, 1‒187.

[B99] Krishnamurthy L. Vadez V. Jyotsna D. M. Serraj R. Nigam S. N. Sheshshayee M. S. (2007). Variation in transpiration efficiency and its related traits in a groundnut (*Arachis hypogaea* L.) mapping population. Field Crops Res. 103, 189–197. 10.1016/j.fcr.2007.06.009

[B100] Kumar S. Thakur P. Kaushal N. Malik J. A. Gaur P. Nayyar H. (2013). Effect of varying high temperatures during reproductive growth on reproductive function, oxidative stress and seed yield in chickpea genotypes differing in heat sensitivity. Archives Agron. Soil Sci. 59 (6), 823–843. 10.1080/03650340.2012.683424

[B101] Lauriano J. A. Lidon F. C. Carvalho C. A. Campos P. S. do Céu Matos M. (2000). Drought effects on membrane lipids and photosynthetic activity in different peanut cultivars. Photosynthetica 38, 7–12. 10.1023/A:1026775319916

[B102] Laxa M. Liebthal M. Telman W. Chibani K. Dietz K. J. (2019). The role of the plant antioxidant system in drought tolerance. Antioxidants 8, 94. 10.3390/antiox8040094 30965652 PMC6523806

[B103] Leal-Bertioli S. C. M. Bertioli D. J. Guimaraes P. M. Pereira T. D. Galhardo I. Silva J. P. (2012). The effect of tetraploidization of wild*Arachis*on leaf morphology and other drought-related traits. Environ. Exp. Bot. 84, 17–24. 10.1016/j.envexpbot.2012.04.005

[B104] Li S. Hu R. Shen G. Zhang H. (2013). Genetic engineering peanut for higher drought- and salt-tolerance. Food Nutr. Sci. 4, 1–7. 10.4236/fns.2013.46a001

[B106] Li J. Li Y. Ma L. (2019). CRISPR/Cas9‐Based genome editing and its applications for functional genomic analyses in plants. Small Methods 3 (3), 1800473. 10.1002/smtd.201800473

[B107] Li H. Yang M. Zhao C. Wang Y. Zhang R. (2021). Physiological and proteomic analyses revealed the response mechanisms of two different drought-resistant maize varieties. BMC Plant Biol. 21, 513. 10.1186/s12870-021-03295-w 34736392 PMC8567644

[B108] Li L. Cui S. Dang P. Yang X. Wei X. Chen K. (2022). GWAS and bulked segregant analysis reveal the Loci controlling growth habit-related traits in cultivated peanut (*Arachis hypogaea* L.). BMC Genom 23, 403. 10.1186/s12864-022-08640-3 PMC9145184 35624420

[B109] Liu X. Z. Hang B. R. (2000). Heat stress injury in relation to membrane lipid peroxidation in creeping bentgrass. Crop Sci. 40, 503–510. 10.2135/cropsci2000.402503x

[B110] Liu X. Kasuga M. Sakuma Y. Abe Miura S. Yamaguchi-Shinozaki K. (1998). Two transcription factors, DREB1 and DREB2, with an EREBP/AP2 DNA binding domain separate two cellular signal transduction pathways in drought- and low-temperature-responsive gene expression, respectively, in Arabidopsis **.** Plant Cell 10, 1391–1406. 10.2307/3870648 9707537 PMC144379

[B111] Luis J. M. Ozias-Akins P. Holbrook C. C. Kemerait R. C. Jr Snider J. L. Liakos V. (2016). Phenotyping peanut genotypes for drought tolerance. Peanut Sci. 43, 36–48. 10.3146/0095-3679-43.1.36

[B112] Lyu J. I. Park J. H. Kim J. K. Bae C. H. Jeong W. J. Min S. R. (2018). Enhanced tolerance to heat stress in transgenic tomato seeds and seedlings overexpressing a trehalose-6-phosphate synthase/phosphatase fusion gene. Plant Biotechnol. Rep. 12, 399–408. 10.1007/s11816-018-0505-8

[B113] Ma W. Zhao T. Li J. Liu B. Fang L. Hu Y. (2016). Identification and characterization of the GhHsp20 gene family in *Gossypium hirsutum* . Sci. Rep. 6, 32517. 10.1038/srep32517 27580529 PMC5007520

[B114] Mallikarjuna N. Senthilvel S. Hoisington D. (2012). Development of new sources of tetraploid Arachis to broaden the genetic base of cultivated groundnut (*Arachis hypogaea* L.). Genet. Resour. Crop Evol. 58, 889–907. 10.1007/s10722-010-9627-8

[B115] Mallikarjuna Swamy B. P. Samia M. Boncodin R. Marundan S. Rebong D. B. Ordonio R. L. (2019). Compositional analysis of genetically engineered GR2E “Golden Rice” in comparison to that of conventional rice. J. Agri. Food Chem. 67, 7986–7994. 10.1021/acs.jafc.9b01524 PMC6646955 31282158

[B116] Mallikarjuna Swamy B. P. Marundan S. Jr. Samia M. Ordonio R. L. Rebong D. B. Miranda R. (2021). Development and characterization of GR2E Golden rice introgression lines. Sci. Rep. 11, 2496. 10.1038/s41598-021-82001-0 33510272 PMC7843986

[B117] Marcum K. B. (1998), Cell membrane thermostability and whole-plant heat tolerance of Kentucky bluegrass. Crop Sci. 38, 1214–1218. 10.2135/cropsci1998.0011183X003800050017x

[B118] Meacham-Hensold K. Fu P. Wu J. Serbin S. Montes C. M. Ainsworth E. (2020). Plot-level rapid screening for photosynthetic parameters using proximal hyperspectral imaging. J. Exp. Bot. 71, 2312–2328. 10.1093/jxb/eraa068 32092145 PMC7134947

[B119] Medrano H. Tomás M. Martorell S. Flexas J. Hernández E. Rosello J. (2015). From leaf to whole-plant water use efficiency (WUE) in complex canopies: Limitations of leaf WUE as a selection target. Crop J. 3 (3), 220–228. 10.1016/j.cj.2015.04.002

[B120] Miller G. Suzuki N. Ciftci-Yilmaz S. Mittler R. (2010). Reactive oxygen species homeostasis and signalling during drought and salinity stresses. Plant Cell Environ. 33, 453–467. 10.1111/j.1365-3040.2009.02041.x 19712065

[B121] Miranda P. V. Iglesias B. F. Charriere M. V. Burachik M. (2022). Drought tolerant wheat IND-ØØ412-7 is nutritionally equivalent to its non-transgenic comparator. Gm. Crops Food 13, 119–125. 10.1080/21645698.2022.2079179 35656970 PMC9176220

[B122] Mittler R. (2002). Oxidative stress, antioxidants and stress tolerance. Trends Plant Sci. 7, 405–410. 10.1016/s1360-1385(02)02312-9 12234732

[B123] Nigam S. N. Dwivedi S. L. Gibbons R. W. (1991). Groundnut breeding: Constraints, achievements and future possibilities. Plant Breed. Abstr. 61 (10), 1127–1136.

[B124] Nigam S. N. Rao R. C. N. Wynne J. C. Williams J. H. Fitzner M. Nagabhushanam G. V. S. (1994). Effect and interaction of temperature and photoperiod on growth and partitioning in three groundnut (*Arachis hypogaea* L.) genotypes^1^ . Ann. Appl. Biol. 125, 541–552. 10.1111/j.1744-7348.1994.tb04991.x

[B125] Nigam S. N. Nageswara Rao R. C. Wynne J. C. (1998). Effects of temperature and photoperiod on vegetative and reproductive growth of groundnut (*Arachis hypogaea* L.). J. Agron. Crop Sci. 181, 117–124. 10.1111/j.1439-037X.1998.tb00406.x

[B126] Nigam S. Chandra S. Sridevi K. R. Bhukta M. Reddy A. G. S. Rachaputi N. R. (2005). Efficiency of physiological trait‐based and empirical selection approaches for drought tolerance in groundnut. Ann. Appl. Biol. 146, 433–439. 10.1111/j.1744-7348.2005.040076.x

[B127] Oladzad A. González A. Macchiavelli R. de Jensen C. E. Beaver J. Porch T. (2020). Genetic factors associated with nodulation and nitrogen derived from atmosphere in a middle American common bean panel. Front. Plant Sci. 11. 10.3389/fpls.2020.576078 PMC7769817 33384700

[B128] Pandey M. K. Monyo E. Ozias-Akins P. Liang X. Guimarães P. Nigam S. N. (2012). Advances in Arachis genomics for peanut improvement. Biotechnol. Adv. 30 (3), 639–651. 10.1016/j.biotechadv.2011.11.001 22094114

[B129] Pandey M. K. Upadhyaya H. D. Rathore A. Vadez V. Sheshshayee M. S. Sriswathi M. (2014). Genome-wide association studies for 50 agronomic traits in peanut using the ‘reference set’comprising 300 genotypes from 48 countries of the semi-arid tropics of the world. PLoS one 9, e105228. 10.1371/journal.pone.0105228 25140620 PMC4139351

[B130] Pandey M. Agarwal G. Kale S. M. Clevenger J. Nayak S. N. Sriswathi M. (2017). Development and evaluation of a high density genotyping 'Axiom_Arachis' array with 58 K SNPs for accelerating genetics and breeding in groundnut. Sci. Rep. 7, 40577 10.1038/srep40577 28091575 PMC5238394

[B131] Pandey M. K. Kumar R. Pandey A. K. Soni P. Gangurde S. S. Sudini H. K. (2019). Mitigating aflatoxin contamination in groundnut through A combination of genetic resistance and post-harvest management practices. Toxins (Basel) 11, 315. 10.3390/toxins11060315 31163657 PMC6628460

[B132] Pandey M. K. Gangurde S. S. Sharma V. Pattanashetti S. K. Naidu G. K. Faye I. (2020). Improved genetic map identified major QTLs for drought tolerance-and iron deficiency tolerance-related traits in groundnut. Genes 12, 37. 10.3390/genes12010037 33396649 PMC7824586

[B133] Pandurangaiah M. Rao L. G. Sudhakarbabu O. Nareshkumar A. Kiranmai K. Lokesh U. (2014). Overexpression of horsegram (*Macrotyloma uniflorum* Lam. Verdc.) NAC transcriptional factor (*MuNAC4*) in groundnut confers enhanced drought tolerance. Mol. Biotechnol. 56, 758–769. 10.1007/s12033-014-9754-0 24748414

[B134] Parmley K. Nagasubramanian K. Sarkar S. Ganapathysubramanian B. Singh A. K. (2019). Development of optimized phenomic predictors for efficient plant breeding decisions using phenomic-assisted selection in soybean. Plant Phenomics 2019, 5809404. 10.34133/2019/5809404 33313530 PMC7706298

[B135] Parsons R. Sunley R. J. (2001). Nitrogen nutrition and the role of root–shoot nitrogen signalling particularly in symbiotic systems. J. Exp. Bot. 52, 435–443. 10.1093/jexbot/52.suppl_1.435 11326050

[B136] Patel K. G. Thankappan R. Mishra G. P. Mandaliya V. B. Kumar A. Dobaria J. R. (2017). Transgenic peanut (*Arachis hypogaea* L.) overexpressing mtlD gene showed improved photosynthetic, physio-biochemical, and yield-parameters under soil-moisture deficit Stress in lysimeter system. Front.Plant Sci. 8, 1881. 10.3389/fpls.2017.01881 29163606 PMC5675886

[B137] Patel J. D. Wang M. L. Dang P. Butts C. Lamb M. Chen C. Y. (2022). Insights into the genomic architecture of seed and pod quality traits in the U.S. Peanut mini-core diversity panel. Plants 11, 837. 10.3390/plants11070837 35406817 PMC9003526

[B138] Patil M. Ramu S. V. Jathish P. Sreevathsa R. Reddy P. C. Ganesh P. (2014). Overexpression of AtNAC2 (ANAC092) in groundnut (*Arachis hypogaea* L.) improves abiotic stress tolerance. Plant Biotechnol. Rep. 8, 161–169. 10.1007/s11816-013-0305-0

[B139] Patrick A. Pelham S. Culbreath A. Holbrook C. C. De Godoy I. J. Li C. (2017). High throughput phenotyping of tomato spot wilt disease in peanuts using unmanned aerial systems and multispectral imaging. IEEE Instru. Meas. Mag. 20, 4–12. 10.1109/MIM.2017.7951684

[B140] Peng Z. Liu F. X. Wang L. P. Zhou H. Paudel D. Tan L. B. (2017). Transcriptome profiles reveal gene regulation of peanut (*Arachis hypogaea* L.) nodulation. Sci. Rep. 7, 40066. 10.1038/srep40066 28059169 PMC5216375

[B141] Peng Z. Chen H. Tan L. Shu H. Varshney R. K. Zhou Z. (2021). Natural polymorphisms in a pair of NSP2 Homoeologs can cause loss of nodulation in peanut. J. Exp. Bot. 72, 1104–1118. 10.1093/jxb/eraa505 33130897

[B142] Peoples M. B. Pate J. S. Atkins C. A. Bergersen F. J. (1986). Nitrogen nutrition and xylem sap composition of peanut (*Arachis hypogaea* L. cv Virginia Bunch) 1. Plant Physiol. 82, 946–951. 10.1104/pp.82.4.946 16665171 PMC1056238

[B143] Pilon C, Snider J. L. Sobolev V. Chastain D. R. Sorensen R. B. Meeks C. D. (2018). Assessing stomatal and non-stomatal limitations to carbon assimilation under progressive drought in peanut (*Arachis hypogaea* L.). J. Plant Physiol. 231, 124–134. 10.1016/j.jplph.2018.09.007 30261481

[B144] Pimratch S. Jogloy S. Vorasoot N. Toomsan B. Patanothai A. Holbrook C. C. (2007). Relationship between biomass production and nitrogen fixation under drought-stress conditions in peanut genotypes with different levels of drought resistance. J. Agron. Crop Sci. 194, 15–25. 10.1111/j.1439-037X.2007.00286.x

[B145] Polania J. A. Poschenrieder C. Beebe S. Rao I. M. (2016). Effective use of water and increased dry matter partitioned to grain contribute to yield of common bean improved for drought resistance. Front. Plant Sci. 7, 660. 10.3389/fpls.2016.00660 27242861 PMC4864351

[B146] Prasad P. V. V. Craufurd P. Q. Summerfield R. J. (1999). Sensitivity of peanut to timing of heat stress during reproductive development. Crop Sci. 39, 1352–1357. 10.2135/cropsci1999.3951352x

[B147] Prasad P. V. V. Boote K. J. Hartwell A. L. Thomas J. M. G. (2003). Super-optimal temperatures are detrimental to peanut (*Arachis hypogaea* L.) reproductive processes and yield at both ambient and elevated carbon dioxide. Glob. Change Biol. 9, 1775–1787. 10.1046/j.1365-2486.2003.00708.x

[B148] Pruthvi V. Narasimhan R. Nataraja K. N. (2014). Simultaneous expression of abiotic stress responsive transcription factors, *AtDREB2A, AtHB7* and *AtABF3* improves salinity and drought tolerance in peanut (*Arachis hypogaea* L.). PLoS ONE 9, e111152. 10.1371/journal.pone.0111152 25474740 PMC4256372

[B149] Qin H. Gu Q. Zhang J. Sun L. Kuppu S. Zhang Y. (2011). Regulated expression of an isopentenyltransferase gene (*IPT*) in peanut significantly improves drought tolerance and increases yield under field conditions. Plant Cell Physiol. 52, 1904–1914. 10.1093/pcp/pcr125 21920877

[B150] Quilambo O. A. (2004). Proline content, water retention capability and cell membrane integrity as parameters for drought tolerance in two peanut cultivars. South Afr. J. Bot. 70, 227–234. 10.1016/S0254-6299(15)30239-8

[B151] Rachaputi N. C. (2003). “Environmental characterization of experimental sites in India and Australia,”. Report of a workshop held at ICRISAT Center, Andhra Pradesh, India, 25-27 February 2002 in Breeding for drought-resistant peanuts. Editors Cruickshank A. W. Rachaputi N. C. Wright G. C. Nigam S. N., 61–66. ACIAR Proceedings No. 112.

[B152] Raj R. Walker J. P. Vinod V. Pingale R. Naik B. Jagarlapudi A. (2021). Leaf water content estimation using top-of-canopy airborne hyperspectral data. Int. J. Appl. Earth Obs. Geoinf. 102, 102393. 10.1016/j.jag.2021.102393

[B153] Ramu V. S. Swetha T. N. Sheela S. H. Babitha C. K. Rohini S. Reddy M. K. (2016). Simultaneous expression of regulatory genes associated with specific drought-adaptive traits improves drought adaptation in peanut. Plant Biotechnol. J. 14, 1008–1020. 10.1111/pbi.12461 26383697 PMC11388866

[B155] Ratnakumar P. Vadez V. (2011). Groundnut (*Arachis hypogaea*) genotypes tolerant to intermittent drought maintain a high harvest index and have small leaf canopy under stress. Funct. Plant Biol. 38, 1016–1023. 10.1071/FP11145 32480959

[B156] Ravi K. Vadez V. Isobe S. Mir R. R. Guo Y. Nigam S. N. (2011). Identification of several small main-effect QTLs and a large number of epistatic QTLs for drought tolerance related traits in groundnut (*Arachis hypogaea* L.). Theor. Appl. Genet. 122, 1119–1132. 10.1007/s00122-010-1517-0 21191568 PMC3057011

[B157] Raza A. Sharif Y. Chen K. Wang L. Fu H. Zhuang Y. (2022). Genome-wide characterization of ascorbate peroxidase gene family in peanut (*Arachis hypogea* L.) revealed their crucial role in growth and multiple stress tolerance. Front. Plant Sci. 13, 962182. 10.3389/fpls.2022.962182 36186077 PMC9524023

[B158] Rebetzke G. J. Condon A. G. Richards R. A. Farquhar G. D. (2002). Selection for reduced carbon isotope discrimination increases aerial biomass and grain yield of rainfed bred wheat. Crop Sci. 42, 739–745. 10.2135/cropsci2002.0739

[B159] Reddy T. Y. Reddy V. R. Anbumozhi V. (2003). Physiological responses of groundnut (*Arachis hypogaea* L.) to drought stress and its amelioration: A critical review. Plant Growth Regul. 41, 75–88. 10.1556/aagr.51.2003.2.9

[B160] Rehman T. U. Ma D. Wang L. Zhang L. Jin J. (2020). Predictive spectral analysis using an end-to-end deep model from hyperspectral images for high-throughput plant phenotyping. Comp. Electron. Agric. 177, 105713. 10.1016/j.compag.2020.105713

[B240] Riley K. (2015). NOAA National Centers for Environmental Information, Monthly Drought Report for Annual 2015, published online January 2016. Available at: https://www.ncei.noaa.gov/access/monitoring/monthly-report/drought/201513 .

[B161] Ribaut J.-M. Betran J. Monneveux P. Setter T. (2009). “Drought tolerance in maize,” in Handbook of maize: Its biology. Editors Bennetzen J. L. Hake S. C. (New York, NY: Springer), 311–344.

[B162] Richards R. A. Rebetzke G. J. Condon A. G. van Herwaarden A. F. (2002). Breeding opportunities for increasing the efficiency of water use and crop yield in temperate cereals. Crop Sci. 42, 111–121. 10.2135/cropsci2002.0111 11756261

[B163] Rincent R. Charpentier J. P. Faivre-Rampant P. Paux E. Le Gouis J. Bastien C. (2018). Phenomic selection is a low-cost and high-throughput method based on indirect predictions: Proof of concept on wheat and poplar. Genes|Genomes|Genetics 8, 3961–3972. 10.1534/g3.118.200760 30373914 PMC6288839

[B164] Rowland D. L. Faircloth W. H. Payton P. Tissue D. T. Ferrell J. A. Sorensen R. B. (2012a). Primed acclimation of cultivated peanut (*Arachis hypogaea* L.) through the use of deficit irrigation timed to crop developmental periods. Agric. Water Manag. 113, 85–95. 10.1016/j.agwat.2012.06.023

[B165] Rowland D. Puppala N. Beasley J. Burow M. Gorbet D. Jorden D. (2012b). Variation in carbon isotope ratio and its relation to other traits in peanut breeding lines and cultivars from U.S. Trials. J. Plant Breed. Crop Sci. 4, 144–155. 10.5897/JPBCS12.031

[B166] Rustgi S. Kakati J. P. Jones Z. T. Zoong Lwe Z. S. Narayanan S. (2021). Heat tolerance as a function of membrane lipid remodeling in the major US oilseed crops (soybean and peanut). J. Plant Biochem. Biotechnol. 30, 652–667. 10.1007/s13562-021-00729-2

[B167] Sakuma Y. Maruyama K. Qin F. Osakabe Y. Shinozaki K. Yamaguchi-Shinozaki K. (2006). Dual function of an Arabidopsis transcription factor DREB2A in water-stress-responsive and heat-stress-responsive gene expression. Proc. Natl. Acad. Sci. U. S. A. 103, 18822–18827. 10.1073/pnas.0605639103 17030801 PMC1693746

[B168] Samanta M. K. Dey A. Gayen S. (2016). CRISPR/Cas9: An advanced tool for editing plant genomes. Trans. Res. 25, 561–573. 10.1007/s11248-016-9953-5 27012546

[B169] Sanz‐Saez A. Maw M. J. W. Polania J. A. Rao I. M. Beebe S. E. Fritschi F. B. (2019). Using carbon isotope discrimination to assess genotypic differences in drought resistance of parental lines of common bean. Crop Sci. 59, 2153–2166. 10.2135/cropsci2019.02.0085

[B170] Sarić R. Nguyen V. D. Burge T. Berkowitz O. Trtílek M. Whelan J. (2022). Applications of hyperspectral imaging in plant phenotyping. Trends Plant Sci. 27 (3), 301–315. 10.1016/j.tplants.2021.12.003 34998690

[B171] Sarkar T. Thankappan R. Kumar A. Mishra G. P. Dobaria J. R. (2014). Heterologous expression of the AtDREB1A gene in transgenic peanut-conferred tolerance to drought and salinity stresses. PLoS ONE 9 (12), e110507. 10.1371/journal.pone.0110507 25545786 PMC4278701

[B172] Sarkar S. Cazenave A. B. Oakes J. McCall D. Thomason W. Abbot L. (2020). High‐throughput measurement of peanut canopy height using digital surface models. Plant Phenome J. 3, e20003. 10.1002/ppj2.20003

[B173] Sarkar S. Cazenave A. B. Oakes J. McCall D. Thomason W. Abbott L. (2021). Aerial high-throughput phenotyping of peanut leaf area index and lateral growth. Sci. Rep. 11, 21661. 10.1038/s41598-021-00936-w 34737338 PMC8569151

[B174] Scarpeci T. E. Zanor M. I. Carrillo N. Mueller-Roeber B. Valle E. M. (2008). Generation of superoxide anion in chloroplasts of *Arabidopsis thaliana* during active photosynthesis: A focus on rapidly induced genes. PlantMol. Biol. 66, 361–378. 10.1007/s11103-007-9274-4 PMC2758387 18158584

[B175] Scheben A. Wolter F. Batley J. Puchta H. Edwards D. (2017). Towards CRISPR/Cas crops–bringing together genomics and genome editing. New Phytol. 216, 682–698. 10.1111/nph.14702 28762506

[B176] Selvaraj G. M. Narayana M. Schubert A. M. Ayers J. L. Baring M. R. Burow M. D. (2009). Identification of QTLs for pod and kernel traits in cultivated peanut by bulked segregant analysis. Electron. J. Biotechnol. 12 (2), 3–4.

[B177] Selvaraj M. G. Burow G. Burke J. J. Belamker V. Puppala N. Burow M. D. (2011). Heat stress screening of peanut (*Arachis hypogaea* L.) seedlings for acquired thermotolerance. Plant Growth Regul. 65, 83–91. 10.1007/s10725-011-9577-y

[B178] Shah Z. Iqbal A. Khan F. U. Khan H. U. Durrani F. Ahmad M. Z. (2020). Genetic manipulation of pea (Pisum sativum L.) with Arabidopsis’s heat shock factor HsfA1d improves ROS scavenging system to confront thermal stress. Genet. Resour. Crop Evol. 67, 2119–2127. 10.1007/s10722-020-00966-9

[B179] Shaibu A. S. Sneller C. Motagi B. N. Chepkoech J. Chepngetich M. Miko Z. L. (2020). Genome-wide detection of SNP markers associated with four physiological traits in groundnut (*Arachis hypogaea* L.) mini core collection. Agronomy 10, 192. 10.3390/agronomy10020192

[B180] Sharma K. K. Anjaiah V. (2000). An efficient method for the production of transgenic plants of peanut (*Arachis hypogaea* L.) through *Agrobacterium tumefaciens*-mediated genetic transformation. Plant Sci. 159, 7–19. 10.1016/s0168-9452(00)00294-6 11011088

[B181] Sharma K. K. Ortiz R. (2000). Program for the application of genetic transformation for crop improvement in the semi-arid tropics. Vitro Cell. Dev. Biol. Plant 36, 83–92. 10.1007/s11627-000-0019-1

[B182] Sharma K. K. Pothana A. Prasad K. Shan D. Kaur J. Bhatnagar D. (2018). Peanuts that keep aflatoxin at bay: A threshold that matters. Plant Biotechnol. J. 16, 1024–1033. 10.1111/pbi.12846 28973784 PMC5902767

[B183] Sharma S. (2017). Prebreeding using wild species for genetic enhancement of grain legumes at ICRISAT. Crop Sci. 57, 1132–1144. 10.2135/cropsci2017.01.0033

[B184] Shekoofa A. Rosas-Anderson P. Sinclair T. R. Balota M. Isleib T. G. (2015). Measurement of limited-transpiration under high vapor pressure deficit for peanut in chambers and in field. Agron. J. 107, 1019–1024. 10.2134/agronj14.0570

[B185] Sinclair T. R. Leilah A. A. Schreffler A. K. (1995). Peanut nitrogen fixation (C2H2 reduction) response to soil dehydration. Peanut Sci. 22, 162–166. 10.3146/i0095-3679-22-2-17

[B186] Sinclair T. Devi J. Shekoofa A. Choudhary S. Sadok W. Vadez V. (2017). Limited transpiration response to high vapor pressuredDeficit in crop ppecies. J. Plant Sci. 260, 109–118. 10.1016/j.plantsci.2017.04.007 28554468

[B187] Sinclair T. R. (2000). Model analysis of plant traits leading to prolonged crop survival during severe drought. Field Crops Res. 68, 211–217. 10.1016/S0378-4290(00)00125-8

[B188] Sinclair T. R. (2011). Challenges in breeding for yield increase for drought. Trends Plant Sci. 16, 289–293. 10.1016/j.tplants.2011.02.008 21419688

[B189] Singh J. Thakur J. K. (2018). “Photosynthesis and abiotic stress in plants,” in Biotic and abiotic stress tolerance in plants (Singapore: Springer), 27–46.

[B190] Songsri P. Jogloy S. Kesmala T. Vorasoot N. Akkasaeng C. Patanothai A. (2008). Heritability of drought resistance traits and correlation of drought resistance and agronomic traits in peanut. Crop Sci. 48, 2245–2253. 10.2135/cropsci2008.04.0228

[B191] Srinivasan A. Takeda H. Senboku T. (1996). Heat tolerance in food legumes as evaluated by cell membrane thermostability and chlorophyll fluorescence techniques. Euphytica 88, 35–45. 10.1007/bf00029263

[B192] Stansell J. R. Pallas J. E. Jr. (1985). Yield and quality response of florunner peanut to applied drought at several growth Stages1. Peanut Sci. 12, 64–70. 10.3146/pnut.12.2.0005

[B193] Steketee C. J. Sinclair T. R. Riar M. K. Schapaugh W. T. Li Z. (2019). Unraveling the genetic architecture for carbon and nitrogen related traits and leaf hydraulic conductance in soybean using genome-wide association analyses. BMC Genom 20, 811. 10.1186/s12864-019-6170-7 PMC6836393 31694528

[B194] Sukanth B. S. (2022). Molecular analysis of physiological and productivity traits under high-temperature stress in groundnut (*Arahis hypogaea* L.). MSc thesis. Dharwad, India: University of Agricultural Sciences.

[B195] Tardieu F. Tuberosa R. (2010)). Dissection and modelling of abiotic stress tolerance in plants. Curr. Opin. Plant Biol. 13 (2), 206–212. 10.1016/j.pbi.2009.12.012 20097596

[B196] Thoppurathu F. J. Ghorbanzadeh Z. Vala A. K. Hamid R. Joshi M. (2022). Unravelling the treasure trove of drought-responsive genes in wild-type peanut through transcriptomics and physiological analyses of root. Funct. Integr. Genom. 22, 215–233. 10.1007/s10142-022-00833-z 35195841

[B197] U.S. Department of Agriculture Agricultural Research Service. (2019), Available at: https://www.ars.usda.gov/oc/fnrb/2019/

[B198] Upadhyaya H. D. Ortiz R. (2001). A mini core subset for capturing diversity and promoting utilization of chickpea genetic resources in crop improvement. Theor. Appl. Genet. 102, 1292–1298.

[B199] Upadhyaya H. D. Bramel P. J. Ortiz R. Singh S. (2002). Geographical patterns of diversity for morphological and agronomic traits in the groundnut germplasm collection. Euphytica 128, 191–204. 10.1023/A:1020835419262

[B200] Upadhyaya H. D. Bramel P. J. Ortiz R. Singh S. (2002a). Developing a mini core of peanut for utilization of genetic resources. Crop Sci. 42, 2150–2156. 10.2135/cropsci2002.2150

[B201] Upadhyaya H. D. Ortiz R. Bramel P. J. Singh S. (2002b). Development of a groundnut core collection using taxonomical, and morphological descriptors. Genet. Resour. Crop. Evol. 50, 39–148. 10.1023/a:1022945715628

[B202] Upadhyaya H. D. (2005). Variability for drought resistance related traits in the mini core collection 0of peanut. Crop Sci. 45, 1432–1440. 10.2135/cropsci2004.0389

[B203] Vadez V. Ratnakumar P. (2016). High transpiration efficiency increases pod yield under intermittent drought in dry and hot atmospheric conditions but less so under wetter and cooler conditions in groundnut (*Arachis hypogaea* (L.)). Field Crops Res. 193, 16–23. 10.1016/j.fcr.2016.03.001 27375341 PMC4896115

[B204] Vara Prasad P. V. Craufurd P. Q. Kakani V. G. Wheeler T. R. Boote K. J. (2001). Influence of temperature during pre- and post anthesis stages of floral development on fruit-set and pollen germination in peanut. Aust. J. Plant Physiol. 28, 233–240.

[B205] Varshney R. K. Pandey M. K. Janila P. Nigam S. N. Sudini H. Gowda M. V. C. (2014). Marker-assisted introgression of a QTL region to improve rust resistance in three elite and popular varieties of peanut (*Arachis hypogaea* L.). Theor. Appl. Genet. 127, 1771–1781. 10.1007/s00122-014-2338-3 24927821 PMC4110420

[B206] Varshney R. K. (2016). Exciting journey of 10 years from genomes to fields and markets: Some success stories of genomics-assisted breeding in chickpea, pigeonpea and groundnut. Plant Sci. 242, 98–107. 10.1016/j.plantsci.2015.09.009 26566828

[B207] Vemanna R. S. Chandrashekar B. K. Rao H. M. H. Sathyanarayanagupta S. K. Sarangi K. S. Nataraja K. N. (2013). A modified multisite gateway cloning strategy for consolidation of genes in plants. Mol. Biotechnol. 53, 129–138. 10.1007/s12033-012-9499-6 22274939

[B208] Venkatesh B. Reddy V. A. Lokesh U. Kiranmai K. Johnson A. A. M. Pandurangaiah M. (2018). Multigenic groundnut transgenics: An advantage over traditional single gene traits in conferring abiotic stress tolerance: A review. J. Agri. Allied Sci. 7, 113–120.

[B209] Venkatesh B. Vennapusa A. R. Kumar N. J. Jayamma N. Reddy B. M. Johnson A. M. A. (2022). Co-expression of stress-responsive regulatory genes, MuNAC4, MuWRKY3 and MuMYB96 associated with resistant-traits improves drought adaptation in transgenic groundnut (*Arachis hypogaea* L.) plants. Front. Plant Sci. 13, 1055851. 10.3389/fpls.2022.1055851 36466254 PMC9709484

[B210] Vessey J. K. Pawlowski K. Bergman B. (2005). Root-based N_2_-fixing symbioses: Legumes, actinorhizal plants, Parasponia sp. and cycads. Plant Soil 274, 51–78. 10.1007/s11104-005-5881-5

[B211] Waltz E. (2016). Gene-edited CRISPR mushroom escapes US regulation. Nature 532, 293. 10.1038/nature.2016.19754 27111611

[B212] Wang C. Wan Y. Liu F. Zhang K. (2018). Study on drought resistance of peanut varieties at seedling stage under PEG6000 osmotic stress. Shandong Agric. Sci. 50, 65–71.

[B213] Wang Y. Wisniewski M. Meilan R. Cui M. Fuchigami L. (2006). Transgenic tomato (Lycopersicon esculentum) overexpressing cAPX exhibits enhanced tolerance to UV-B and heat stress. J. Appl. Hortic. 8, 87–90. 10.37855/jah.2006.v08i02.21

[B214] Wang P. Song H. Li C. Li P. Li A. Guan H. (2017). Genome-wide dissection of the heat shock transcription factor family genes in Arachis. Front. Plant Sci. 8, 106. 10.3389/fpls.2017.00106 28220134 PMC5292572

[B215] Wang W. Wang W. Pan Y. Tan C. Li H. Chen Y. (2022). A new gain-of-function OsGS2/GRF4 allele generated by CRISPR/Cas9 genome editing increases rice grain size and yield. Crop J 10, 1207–1212. 10.1016/j.cj.2022.01.004

[B216] Wang H. Lei Y. Wan L. Yan L. Lv J. Dai X. (2016). Comparative transcript profiling of resistant and susceptible peanut post-harvest seeds in response to aflatoxin production by *Aspergillus flavus* . BMC Plant Biol. 16, 54. 10.1186/s12870-016-0738-z 26922489 PMC4769821

[B217] Wang H. Qian X. Zhang L. Xu S. Li H. Xia X. (2018). A method of high throughput monitoring crop physiology using chlorophyll fluorescence and multispectral imaging. Front. Plant Sci. 9, 407. 10.3389/fpls.2018.00407 29643864 PMC5883069

[B218] Wang M. L. Grusak M. A. Chen C. Y. Tonnis B. Barkley N. A. Evans E. (2016). Seed protein percentage and mineral concentration variability and their correlation with other seed quality traits in the U.S. peanut mini-core collection. Peanut Sci. 43, 119–125. 10.3146/PS15-15.1

[B219] Wang Q. L. Chen J. H. He N. Y. Guo F. Q. (2018). Metabolic reprogramming in chloroplasts under heat stress in plants. Int. J. Mol. Sci. 19, 849. 10.3390/ijms19030849 29538307 PMC5877710

[B220] Weiß T. M. Zhu X. Leiser W. L. Li D. Liu W. Schipprack W. (2022). Unraveling the potential of phenomic selection within and among diverse breeding material of maize (*Zea mays* L.). G3 Genes, Genomes, Genet. 12, jkab445. 10.1093/g3journal/jkab445 PMC8895988 35100379

[B221] White J. W. Castillo J. A. Ehleringer J. (1990). Associations between productivity, root growth and carbon isotope discrimination in *Phaseolus vulgaris* under water deficit. Funct. Plant Biol. 17, 189–198. 10.1071/pp9900189

[B222] Wright G. C. Rao R. C. N. Farquhar G. D. (1994). Water-use efficiency and carbon isotope discrimination in peanut under water deficit conditions. Crop Sci. 34, 92–97. 10.2135/cropsci1994.0011183X003400010016x

[B223] Xie K. Minkenberg B. Yang Y. (2015). Boosting CRISPR/Cas9 multiplex editing capability with the endogenous tRNA-processing system. PNAS 112, 3570–3575. 10.1073/pnas.1420294112 25733849 PMC4371917

[B224] Xu W. Yu G. Zare A. Zurweller B. Rowland D. L. Reyes-Cabrera J. (2020). Overcoming small minirhizotron datasets using transfer learning. Comp. Electron. Agric. 175, 105466. 10.1016/j.compag.2020.105466

[B225] Yang X. Luo L. Yu W. Mo B. Liu L. (2019). Recent advances in the acclimation mechanisms and genetic improvement of peanut for drought tolerance. Agric. Sci. 10, 1178–1193. 10.4236/as.2019.109088

[B226] Yao J. Sun D. Cen H. Xu H. Weng H. Yuan F. (2018). Phenotyping of *Arabidopsis* drought stress response using kinetic chlorophyll fluorescence and multicolor fluorescence imaging. Front. Plant Sci. 9, 603. 10.3389/fpls.2018.00603 29868063 PMC5958224

[B227] Yeh D. M. Lin H. F. (2003). Thermostability of cell membranes as a measure of heat tolerance and relationship to flowering delay in chrysanthemum. J. Am. Soc. Hortic. Sci. 128, 656–660. 10.21273/JASHS.128.5.0656

[B228] Yuan H. Bennett R. S. Wang N. Chamberlin K. D. (2019). Development of a peanut canopy measurement system using a ground-based lidar sensor. Front. Plant Sci. 10, 203. 10.3389/fpls.2019.00203 30873193 PMC6403138

[B229] Zhang Y. Xia H. Yuan M. Zhao C. Li A. Wang X. (2011). Cloning and expression analysis of peanut (*Arachis hypogaea* L.) CHI gene. Electron. J. Biotechnol. 15, 9–12. 10.2225/vol15-issue1-fulltext-6

[B230] Zhang J. Liu B. Zhang L. Wang Y. Zheng H. (2015). *Hsf* and *hsp* gene families in *populus*: Genome-wide identification, organization and correlated expression during development and in stress responses. BMC Genomics 16, 181. 10.1186/s12864-015-1398-3 25887520 PMC4373061

[B231] Zhang Q. Dang P. Chen C. Feng Y. Batchelor W. Lamb M. (2022). Tolerance to mid-season drought in peanut can be achieved by high water use efficiency or high efficient use of water. Crop Sci. 62, 1948–1966. 10.1002/csc2.20806

[B232] Zhang H. Wang M. L. Schaefer R. Dang P. Jiang T. Chen C. (2019). GWAS and coexpression network reveal ionomic variation in cultivated peanut. J. Agric. Food Chem. 67, 12026–12036. 10.1021/acs.jafc.9b04939, 31589432

[B234] Zhao Y. Chan Z. Gao J. Xing L. Cao M. Chunmei Y. (2016). ABA receptor PYL9 promotes drought resistance and leaf senescence. Proc. Natl. Acad. Sci. 113, 1949–1954. 10.1073/pnas.1522840113 26831097 PMC4763734

[B235] Zhu X. Maurer H. P. Jenz M. Hahn V. Ruckelshausen A. Leiser W. L. (2022). The performance of phenomic selection depends on the genetic architecture of the target trait. Theor. Appl. Genet. 135, 653–665. 10.1007/s00122-021-03997-7 34807268 PMC8866387

[B236] Zoong Lwe Z. S. Welti R. Anco D. Rustgi S. Narayanan S. (2020). Heat stress elicits remodeling in the anther lipidome of peanut. Sci. Rep. 10, 22163. 10.1038/s41598-020-78695-3 33335149 PMC7747596

[B237] Zou K. Kim K.-S. Kang D. Kim M.-C. Ha J. Moon J.-K. (2022). Genome-wide association study of leaf chlorophyll content using high-density SNP array in peanuts (*Arachis hypogaea* L.). Agronomy 12, 152. 10.3390/agronomy12010152

[B238] Zurweller B. A. Rowland D. L. Tillman B. L. Payton P. P. Migliaccio K. Wright D. (2018). Assessing above- and below-ground traits of disparate peanut genotypes for determining adaptability to soil hydrologic conditions. Field Crops Res. 219, 98–105. 10.1016/j.fcr.2018.01.020

